# Short-Term Erythropoietin Treatment Does Not Substantially Modulate Monocyte Transcriptomes of Patients with Combined Heart and Renal Failure

**DOI:** 10.1371/journal.pone.0041339

**Published:** 2012-09-05

**Authors:** Kim E. Jie, Karien van der Putten, Sebastiaan Wesseling, Jaap A. Joles, Marloes W. Bergevoet, Floor Pepers-de Kort, Pieter A. Doevendans, Yutaka Yasui, Qi Liu, Marianne C. Verhaar, Carlo A. Gaillard, Branko Braam

**Affiliations:** 1 Department of Nephrology and Hypertension, University Medical Center Utrecht, Utrecht, The Netherlands; 2 Department of Internal Medicine, Leiden University Medical Center, Leiden, The Netherlands; 3 Department of Internal Medicine, Meander Medical Center Amersfoort, Amersfoort, The Netherlands; 4 Service XS B.V., Leiden, The Netherlands; 5 Department of Cardiology, University Medical Center Utrecht, Utrecht, The Netherlands; 6 Department of Public Health Sciences, School of Public Health, University of Alberta, Edmonton, Canada; 7 Department of Nephrology, Vrije Universiteit Medical Center, Amsterdam, The Netherlands; 8 Department of Medicine, Division of Nephrology and Immunology, University of Alberta, Edmonton, Canada; 9 Department of Physiology, University of Alberta, Edmonton, Canada; Center of Ophtalmology, Germany

## Abstract

**Background:**

Combined heart and renal failure is associated with high cardiovascular morbidity and mortality. Anti-oxidant and anti-inflammatory, non-hematopoietic effects of erythropoietin (EPO) treatment have been proposed. Monocytes may act as biosensors of the systemic environment. We hypothesized that monocyte transcriptomes of patients with cardiorenal syndrome (CRS) reflect the pathophysiology of the CRS and respond to short-term EPO treatment at a recommended dose for treatment of renal anemia.

**Methods:**

Patients with CRS and anemia (n = 18) included in the EPOCARES trial were matched to healthy controls (n = 12). Patients were randomized to receive 50 IU/kg/week EPO or not. RNA from CD14^+^-monocytes was subjected to genome wide expression analysis (Illumina) at baseline and 18 days (3 EPO injections) after enrolment. Transcriptomes from patients were compared to healthy controls and effect of EPO treatment was evaluated within patients.

**Results:**

In CRS patients, expression of 471 genes, including inflammation and oxidative stress related genes was different from healthy controls. Cluster analysis did not separate patients from healthy controls. The 6 patients with the highest hsCRP levels had more differentially expressed genes than the 6 patients with the lowest hsCRP levels. Analysis of the variation in log_2_ ratios of all individual 18 patients indicated that 4 of the 18 patients were different from the controls, whereas the other 14 were quite similar. After short-term EPO treatment, every patient clustered to his or her own baseline transcriptome. Two week EPO administration only marginally affected expression profiles on average, however, individual gene responses were variable.

**Conclusions:**

In stable, treated CRS patients with mild anemia, monocyte transcriptomes were modestly altered, and indicated imprints of inflammation and oxidative stress. EPO treatment with a fixed dose has hematopoietic effects, had no appreciable beneficial actions on monocyte transcription profiles, however, could also not be associated with undesirable transcriptional responses.

## Introduction

Patients suffering from chronic heart failure (CHF) and concomitant renal failure have increased cardiovascular morbidity and mortality [1]. Conversely, chronic kidney disease (CKD) patients have an increased risk for myocardial infarction with higher mortality compared to the general population [2]. This condition in which combined cardiac and renal dysfunction aggravates failure of the individual organs has been described as the cardiorenal syndrome (CRS) [3]. In this paper, CRS is defined as the combination of CHF and CKD. Among the pathways involved in the pathogenesis of CRS are oxidative stress, inflammation, the renin-angiotensin system (RAS) and the sympathetic nervous system (SNS), the cardiorenal connectors [3].

Anemia is a well-recognized problem in chronic renal disease. The pathophysiology of renal anemia includes an absolute and/or relative deficiency to erythropoietin (EPO) and a reduced sensitivity to EPO of red-cell lineages. Regarding the former, analogues of the human EPO are available to increase EPO levels. Regarding the latter, transferrin receptor-bound polymeric IgA1 was recently identified as an important modulator of the bone marrow response to EPO [4]. Besides the cells involved in erythropoiesis, a number of other cells involved in cardiovascular disease have been shown to express EPO receptors (reviewed in [5]) including monocytes [6].

Anemia can aggravate heart and renal failure and is associated with worse outcome in CHF [7] and CKD [8]. EPO can be used to treat renal anemia, however, normalization of hemoglobin (Hgb) in CKD patients is not associated with improved cardiovascular outcome [9], [10], [11]. In contrast, high EPO doses and the inability to reach target Hgb seem to be associated with adverse outcome [12]. EPO also has non-hematopoietic, anti-inflammatory and anti-oxidative effects on kidneys, brain, heart and vasculature [13], [14]. Nonetheless, not all reports are equivocal, and undesirable actions such as oxidative stress have also been reported [5].

Among several other circulating cells, monocytes can be used as sensors for direct EPO effects on these cells or for indirect actions of EPO by affecting the internal environment. Monocytes have previously been shown to be able to serve as biosensors to detect changes in the systemic environment [15] and to evaluate the response to treatment [16], [17]. In addition, monocytes are key players in the initiation and progression of atherosclerosis. Monocytes have shown to be sensitive to several of the cardiorenal connectors, such as cytokines and angiotensin II [18], [19]. We have recently described that monocytes from CKD patients display increased expression of genes coding for suppressors of cytokine signaling proteins [20], [21]. Monocyte transcriptomes also correlated with collateral artery formation in patients with coronary artery disease [22].

We have previously shown that short-term, low dose EPO treatment increases erythropoiesis as detected by increased reticulocyte counts [24]. In the present study, it was investigated whether monocyte gene expression profiles of cardiorenal patients reflect the altered systemic environment in CRS and are responsive to short-term, low-dose EPO treatment. First, we explored whether monocytes of CRS patients indeed display altered gene expression profiles compared to healthy controls. Moreover, we investigated whether short-term EPO treatment affects monocyte gene transcription. Since EPO might have undesirable effects, both potentially beneficial effects of EPO, such as induction of anti-inflammatory and anti-oxidant genes and potential detrimental effects on monocyte transcriptomes were investigated.

## Methods

### Study design

The present study is part of the EPOCARES trial (ClinicalTrials.gov, NCT00356733), in which CRS patients on regular treatment and standardized iron supplementation were randomized to receive fixed dose subcutaneous EPO treatment or no EPO treatment. Details of the study design have previously been described [23], [24]. Matched for age and gender, we selected 18 patients with mild anemia (10.3–11.9 g/dl in women and 10.3–12.6 g/dl in men), moderate renal failure (estimated creatinine clearance 20–70 ml/min calculated by Cockcroft-Gault formula) and CHF. CHF was defined as New York Heart Association class II-IV, based on symptoms, signs and objective abnormality on echocardiography [25]. Patients were included with reduced ejection fraction (<50%) or left ventricular end diastolic volume index <97 ml/m^2^ with evidence of diastolic left ventricular dysfunction [26]. The medical ethics committee of the Univ. Medical Center Utrecht, The Netherlands approved the protocol and all patients gave their written informed consent. All procedures were in accordance with the Helsinki Declaration.

After enrolment, 12 out of 18 patients were randomized to receive a low dose of Epoetin-β therapy (50 IU/kg/wk; Neorecormon, Roche Pharmaceuticals). Dosages of EPO used in cardio- and cerebro-protection are higher than the dosages normally used for the treatment of renal anemia. It should also be remarked, that despite the desire to study higher doses of EPO in this setting, this would not be justifiable, given the data that is out there to suggest that the higher dosages may be associated unwanted cardiovascular effects [9], [11], [27]. Biochemical analysis and monocyte isolation for gene expression analyses were performed at baseline and after 18 days of EPO treatment (i.e. after 3 EPO injections), prior to the expected rise in Hgb level. Twelve healthy age- and gender-matched persons served as baseline controls.

### Sample collection and microarray procedures

Peripheral blood was collected from patients and healthy controls in EDTA containing tubes after 30 minutes in a resting position. Blood was kept on ice. Within 3 hours of blood withdrawal, CD14^+^-monocytes were positively isolated with the use of immunomagnetic beads (Invitrogen, CA). The purity of the isolated monocyte population was on average 90% as determined by CD14^+^-staining on flow cytometry analysis.

mRNA was isolated from cell collections using Trizol reagent (Invitrogen/Gibco, CA) according to the manufacturer's instruction. Subsequently, mRNA was purified with NucleoSpin® RNAII (Macherey-Nagel, Düren, Germany) and samples were sent to ServiceXS (Leiden, The Netherlands) for further microarray processing. In brief, quality and integrity of RNA was checked by lab-on-chip technology (Bioanalyzer Agilent, CA). Subsequently, Illumina TotalPrep RNA Amplificationkit (Applied Biosystems/Ambion, TX) was used to create double-stranded cDNA from 500 ng total RNA. After cDNA purification, in vitro transcription reaction resulted in aRNA, which was also purified. Amplified biotinylated aRNA was finally randomly hybridized to HumanRef-8 V3.0 Expression BeadChips (Illumina, CA), followed by scanning for raw gene expression intensities on Illumina's BeadArray scanner.

### Validation of gene array results by real-time quantitative polymerase chain reaction (qPCR)

Monocyte cDNA samples from all patients before and after 18 days EPO treatment and from healthy controls were subjected to real-time qPCR by BioMark dynamic array technology (Fluidigm, CA), performed at ServiceXS. The cDNA samples were exposed to specific target amplification, using PreAmp Master Mix and Gene Expression Assays (Taqman; Applied Biosystems, TX) for IL8, FOS, EGR1, CX3CR1, ADRB2, EPO-R and housekeeping genes GAPDH, 18S, β-Actin and RPL13a. They were subjected to a BioMark dynamic array for determination of Ct-values. Each gene was measured in triplicate for each sample. The default Taqman PCR protocol was used with an annealing temperature of 60°C and a total of 35 cycles of PCR.

### Statistical analyses

Clinical characteristics are presented as mean±standard deviation or median (interquartile range) for respectively normally and not normally distributed data. Analysis between groups for statistically significant differences was performed by Student's t-test, Mann-Whitney U test or paired analysis when appropriate. P-values<0.05 were considered significant.

For microarray data analysis, individual bead outliers were removed from raw bead data if signal intensity was higher or lower than median intensity plus or minus 2× median absolute deviation respectively (T4Illumina, software developed by the authors). The transcript level of a gene in each sample was considered present when the average raw intensity of the gene was significantly higher than negative controls from the same BeadArray (t-test; T4Illumina). Genes were significantly present (transcriptionally active) in a group when present in at least 8 or 13 out of 12 or 18, respectively (based on group size by Z-test, SigmaStat). After normalization procedure (Log_2_-Quantile; FlexArray version 1.6 [28]), differential expression of a gene between two groups was tested by unpaired Cyber t-test for the comparison between groups at baseline and paired t-test for the comparison between patients at baseline and after two weeks of EPO treatment [29]. A. P-value<0.05 was considered significant. To investigate the effects of EOP, genes that were significantly differentially expressed were analyzed by hierarchical clustering, with Euclidean distance as a similarity measure and clustering was based on average-linkage correlation (Multi-experiment-Viewer, MeV, version 4.8 [30]). To construct the heat-map, relative expression levels for each gene and each individual were calculated as a ratio between the actual signal for that gene and the average signal for that gene at baseline. Common oxidative stress-, inflammation- and RAS-related genes were specifically addressed to search for differences between healthy controls and patients, and for the effect of short-term EPO treatment in patients. Moreover, because of the relevance of inflammation for monocyte function, we subdivided the group of patients into three tertiles of CRP levels and investigated whether CRP levels were associated with different transcriptomes at baseline by comparing the lowest and highest tertiles. Finally, variation in the normally distributed log_2_ ratios of each of the patients' baseline datasets was individually compared to variation of the control datasets. This provided further insight in the homogeneity of the baselines samples.

For real-time qPCR analysis, software version 2.0.6 was used for Ct determination from the reaction chambers on the array. Linear baseline correction was applied and the Ct threshold method selected was ‘Auto (Global)’. Average Ct values per gene for each sample were calculated for data that passed amplification curve quality thresholds (default value 0.65). GAPDH served as housekeeping gene, since this gene was considered most stable when comparing 18S, β-Actin, RPL13a and GAPDH in both NormFinder and GeNorm. Normalized gene expression (Ct gene of interest–Ct GAPDH; ΔCt) was related to normalized gene expression in the reference group (ΔΔCt). Fold changes were calculated by 2^−ΔΔCT^ [20], [21]. Gene expression differences between healthy controls and patients, and effects of EPO treatment were tested by Student's t-test and paired analyses respectively. P-value<0.05 was considered significant.

### Accession codes

The data discussed in this publication have been deposited in MIAME compliant NCBI's Gene Expression Omnibus [31] and are accessible through GEO Series accession number GSE17582 (http://www.ncbi.nlm.nih.gov/geo/query/acc.cgi?acc=GSE17582).

## Results

### Study population characteristics

Baseline characteristics of patients with CRS and anemia and age- and gender-matched controls are described in table 1. After the baseline measurement, twelve out of 18 patients were started on EPO treatment. After 18 days, Hgb was not increased significantly compared to baseline (delta Hgb 0.5±1.0 g/dl in EPO-treated group vs. −0.4±1.0 g/dl in non EPO-treated group; p = 0.133). The short-term EPO treatment did trigger the hematopoietic system as indicated by increased reticulocyte counts (0.05±0.02 vs. 0.07±0.02×10e12/l; p<0.001). Whole blood mononuclear cell (MNC) counts were not different in patients compared to healthy controls (6.3(3.2) vs. 5.1(2.0)×10^6^ MNC/ml whole blood; p = 0.215) and remained unchanged after short-term EPO treatment (6.3(3.2) vs. 6.8(2.7)×10^6^ MNC/ml whole blood; p = 0.420).

**Table 1:** Baseline characteristics.

	Healthy controls	Patients	P-value
	(n = 12)	(n = 18)	
Age (years)	68±12	70±11	N.S.
Male gender (%)	8 (67%)	12 (67%)	N.S.
Body mass index (kg/m^2^)	23.7±1.9	26.7±4.5	0.019
Estimated creatinine clearance (ml/min)	69±19	36±11	<0.001
Hemoglobin (g/dl)	14.0±0.8	11.8±0.9	<0.001
Total cholesterol (mmol/l)	5.08±1.26	4.39±1.52	N.S.
HDL (mmol/l)	1.39±0.37	1.21±0.29	N.S.
LDL (mmol/l)	3.31±1.07	2.38±1.17	0.039
Triglycerides (mmol/l)	0.84±0.50	1.75±1.70	N.S.
hsCRP (mg/l)	1.0 (0.8)	4.0 (8.0)	0.007
Ejection fraction (%)	-	46±4	
Systolic blood pressure (mmHg)	129±24	129±18	N.S.
Diastolic blood pressure (mmHg)	81±7	70±8	0.001
Diabetes Mellitus (%)	-	6 (33%)	
Smoking (%)	2 (17%)	3 (17%)	N.S.
**Medication**			
- Acetylsalicylic acid (%)	-	8 (44%)	
- Statin (%)	-	10 (56%)	
- Angiotensin blockade (ACEi/ARB) (%)	-	16 (89%)	
-β-blockade (%)	-	13 (72%)	
- Spironolactone (%)	-	4 (22%)	

Values are expressed as mean ± SD, number (percentage) or median (interquartile range).

hsCRP: high sensitive C-reactive protein; ACEi: angiotension-converting enzyme inhibitor; ARB: angiotensin II receptor blocker; N.S. not significant.

### Erythropoietin receptor expression on monocytes

All monocyte samples showed a significant gene expression of the EPO receptor (EPO-R), reflected by Ct values of 18.0±0.5 on real-time qPCR. Slightly higher EPO-R gene expression was found in patients compared to controls (fold change 1.2; p = 0.05). EPO treatment for 18 days did not significantly alter EPO-R expression (fold change 1.0).

### Monocyte gene expression profile in cardiorenal syndrome patients compared to healthy controls

#### Global gene expression profile changes, hierarchical clustering

We compared monocyte gene expression profiles of CRS patients (n = 18) and healthy controls (n = 12) at baseline. Out of 25,528 genes explored, signals of 12,165 exceeded background in at least one of the two groups; 471 genes were differentially expressed in CRS patients vs. healthy controls (p-value<0.05; 1.8% of total assessed genes). Correction for multiple testing, for example by applying FDR, was not performed because of the very small sample size. Fold changes were low, ranging from 0.3–1.7. Cluster analysis of differentially expressed genes did not separate patients from controls. Clustering of genes could also not be explained by gender or age of the investigated subjects.

#### Specific gene expression changes

In CRS patients, 214 genes displayed increased and 257 decreased expression compared to healthy controls. Table 2 gives an overview of the 15 most induced and downregulated genes for this comparison. A table with all genes with changed expression can be found in table S1. Patients showed lower expression of the transcription factors EGR1 and FOS. Furthermore, patients showed decreased expression of hemoglobin-related genes HBA2 and HBB compared to healthy controls. Additional experiments with extra washing steps of isolated monocyte samples suggest that expression of these two genes could possibly be attributed to reticulocyte contamination (data not shown).

**Table 2:** Top 15 of induced and downregulated genes in CRS patients vs. healthy controls.

UPREGULATED (ranked by descending fold change)					
	Accession Number	Symbol	Description	Fold change	P-value
	NM_018487.2	HCA112	Transmembrane protein 176A	1.695	0.037
	NM_012456.1	TIMM10	Translocase of inner mitochondrial membrane 10 homolog	1.591	<0.0001
	NM_017911.1	C22ORF8	Family with sequence similarity 118, member A	1.582	0.012
	NM_001337.3	CX3CR1	Chemokine (C-X3-C motif) receptor 1	1.491	<0.0001
	NM_006498.2	LGALS2	Lectin, galactoside-binding, soluble, 2	1.464	0.028
	NM_000024.3	ADRB2	Adrenergic, β2-, receptor, surface	1.446	0.002
	NM_005771.3	DHRS9	Dehydrogenase/reductase (SDR family) member 9	1.434	0.008
	NM_198097.1	C7ORF28B	Chromosome 7 open reading frame 28B	1.428	0.005
	NM_001343.1	DAB2	Disabled homolog 2, mitogen-responsive phosphoprotein	1.425	<0.001
	NM_016021.2	UBE2J1	Ubiquitin-conjugating enzyme E2, J1	1.424	<0.001
	NM_030670.1	PTPRO	Protein tyrosine phosphatase, receptor type, variant 6	1.385	<0.0001
	NM_001008566.1	TPST2	Tyrosylprotein sulfotransferase 2	1.379	0.001
	NM_030671.1	PTPRO	Protein tyrosine phosphatase, receptor type, variant 5	1.372	<0.001
	NR_003038.1	SNHG5	Small nucleolar RNA host gene 5	1.359	0.037
	NM_080914.1	ASGR2	asialoglycoprotein receptor 2	1.358	0.003
**DOWNREGULATED (ranked by ascending fold change)**					
	NM_000518.4	HBB	Hemoglobin, β	0.297	0.001
	NM_000517.3	HBA2	Hemoglobin, α2	0.376	0.003
	XM_936120.1	HLA-DQA1	PREDICTED: major histocompatibility complex, class II	0.474	0.006
	NM_000584.2	IL8	Interleukin 8	0.584	<0.001
	NM_005252.2	FOS	V-fos FBJ murine osteosarcoma viral oncogene homolog	0.606	0.012
	NM_006732.1	FOSB	FBJ murine osteosarcoma viral oncogene homolog B	0.608	<0.0001
	NM_001964.2	EGR1	Early growth response 1	0.646	0.008
	NM_004417.2	DUSP1	Dual specificity phosphatase 1	0.678	0.003
	NM_004666.1	VNN1	Vanin 1	0.680	0.003
	NM_024933.2	FLJ12056	Ankyrin repeat domain 53	0.692	0.012
	NM_005502.2	ABCA1	ATP-binding cassette, sub-family A, member 1	0.707	0.009
	NM_020152.2	C21ORF7	Chromosome 21 open reading frame 7	0.710	0.009
	NM_017933.3	FLJ20701	Phosphotyrosine interaction domain containing 1	0.725	<0.001
	NM_002612.2	PDK4	Pyruvate dehydrogenase kinase, isozyme 4	0.736	0.017
	NM_021732.1	AVPI1	Arginine vasopressin-induced 1	0.737	0.006

**Figure 1:**
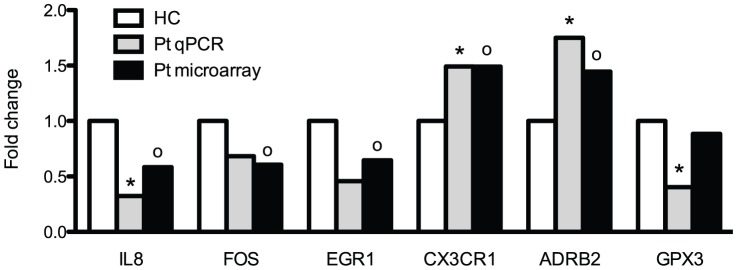
Validation of differentially expressed genes on microarray by quantitative polymerase chain reaction. *P-value<0.05 for gene expression in patients at baseline vs. healthy controls as determined with qPCR technique; ^o^P-value<0.05 for gene expression in patients at baseline vs. healthy controls as determined with microarray technique. ADRB2 adrenergic receptor, β2; CX3CR1 chemokine (C-X3-C motif) receptor 1; EGR1 early growth response 1; FOS FBJ murine osteosarcoma viral oncogene homolog; GPX3 glutathione peroxidase 3; IL8 interleukin 8; qPCR quantitative polymerase chain reaction.

We specifically addressed whether inflammation and oxidative stress in CRS patients were reflected by monocyte gene expressions. Several interesting genes involved in inflammation (i.e. IL8, IL17, IL1RAP, CX3CR1, and several TLRs) and oxidative stress (i.e. DUSP1, GPX3, DHRS9) were indeed modulated in CRS patients (table 3, and table S2 for the entire panel of genes). Remarkably, some of these genes exert pro- and others anti-stimulating activities. With regard to SNS, only ADRB2 was induced in patients. Differential expression of IL8, FOS, EGR1, CX3CR1 and ADRB2 was confirmed by qPCR (figure 1).

**Table 3:** Induction and downregulation of oxidative stress and inflammation related genes in CRS patients vs. healthy controls.

Transcript	Symbol	Definition	Fold change	P-value
INFLAMMATION RELATED GENES				
		**Cytokines and cytokine receptors**		
NM_000628.3	IL10RB	Interleukin 10 receptor	1.15	0.044
NM_005535.1	IL12RB1	Interleukin 12 receptor, β1	1.10	0.006
NM_001560.2	IL13RA1	Interleukin 13 receptor, α1	1.15	0.037
NM_014339.3	IL17R	Interleukin 17 receptor	1.19	0.012
NM_004633.3	IL1R2	Interleukin 1 receptor, type II	0.83	0.044
NM_002182.2	IL1RAP	Interleukin 1 receptor accessory protein	0.89	0.017
NM_173842.1	IL1RN	Interleukin 1 receptor antagonist	0.94	*0.052*
NM_181078.1	IL21R	Interleukin 21 receptor	1.10	*0.066*
NM_004843.2	IL27RA	Interleukin 27 receptor, α	1.22	0.001
NM_000584.2	IL8	Interleukin 8	0.58	0.001
NM_001557.2	IL8RB	Interleukin 8 receptor, β	0.87	*0.079*
		**Chemokines and chemokine receptors**		
NM_002982.3	CCL2	Chemokine (C-C motif) ligand 2	0.77	0.008
NM_002983.1	CCL3	Chemokine (C-C motif) ligand 3	0.86	0.027
NM_001001437.3	CCL3L3	Chemokine (C-C motif) ligand 3-like 3	0.77	0.019
NM_001337.3	CX3CR1	Chemokine (C-X3-C motif) receptor 1	1.49	<0.001
		**Inflammatory response**		
NM_003264.3	TLR2	Toll-like receptor 2	1.16	0.015
NM_006068.2	TLR6	Toll-like receptor 6	1.09	0.021
NM_016562.3	TLR7	Toll-like receptor 7	1.20	0.040
		**Interferon transcriptional regulation**		
NM_002200.3	IRF5	Interferon regulatory factor 5	1.44	*0.051*

OXIDATIVE STRESS RELATED GENES				
		**Antioxidants**		
NM_002084.2	GPX3	Glutathione peroxidase 3	0.88	0.017
NM_006793.2	PRDX3	Peroxiredoxin 3	0.85	*0.061*
NM_203472.1	SELS	Selenoprotein S	0.94	*0.060*
NM_005771.3	DHRS9	Dehydrogenase/reductase (SDR family) member 9	1.33	0.006
		**Genes involved in ROS metabolism**		
NM_024505.2	NOX5	NADPH oxidase, EF-hand calcium binding domain 5	1.05	*0.052*
NM_001141.1	ALOX15B	Arachidonate 15-lipoxygenase, second type	0.93	*0.084*
NM_004417.2	DUSP1	Dual specificity phosphatase 1	0.68	0.003

#### Subanalysis with respect to CRP

After dividing the 18 patients in three groups, we compared the 6 patients with the lowest CRP levels with the patients with highest CRP levels; the middle tertile was left out of the comparison. This subanalysis confirmed that CRP does influence monocyte transcriptomes (table 4). The group sizes become too small for a detailed comparison.

**Table 4:** Number of differentially expressed genes at different expression and significance levels in patients with low CRP and high CRP compared to healthy controls at baseline.

CRP	Low	Middle	High
N =	**6**	**6**	**6**
CRP (average, min-max)	**1.2 (1–3)**	**4.2 (3–6)**	**21.7 (7–53)**
Differentially expressed genes vs. controls	**# of genes**		**# of genes**
P<0.05	**717**		**771**
P<0.01	**125**		**192**
P<0.001	**17**		**25**
P<0.0001	**1**		**10**
P<0.05 & FC<0.74 or >1.35	**16**		**58**
P<0.001 & FC<0.82 or >1.2	**8**		**21**
P<0.0001 & FC<0.91 or >1.1	**1**		**10**

#### Subanalysis with respect to individual variation in gene expression of the cardiorenal patients at baseline

Because of the relatively limited differential expression at baseline, we analyzed the homogeneity of the baseline samples of the CRS patients compared to the controls by comparing the standard deviation of the log_2_ ratios. Indeed, 4 out of the 18 patients showed substantial variation in log_2_ ratios whereas the other did show minimal variation. The number was too small to investigate whether the response to EPO of these 4 patients was different from the 14 other patients.

### Monocyte gene expression in cardiorenal syndrome patients after 18 days of currently recommended dose erythropoietin treatment

#### Global gene expression profile changes, hierarchical clustering

**Figure 2:**
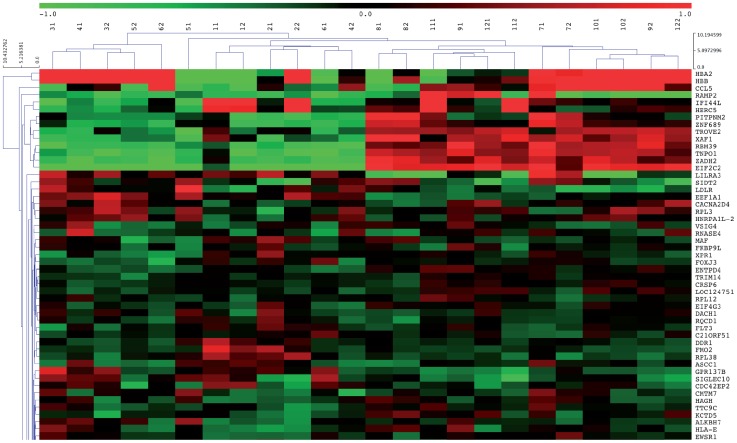
Euclidean cluster analysis for patients before and after erythropoietin treatment. Patient number and time point of sample collection (patient 1, timepoint 1 = 11; patient 1, timepoint 2 = 12, etc) are listed in order of monocyte transcriptomes similarity. The closer samples are depicted to each other, the more comparable transcriptomes are. Only the first 50 genes are depicted, the full figure is online.

The effect of 18 days EPO treatment was assessed in the 12 CRS patients before and after the initiation of EPO treatment. Out of 25,528 genes explored, signals of 12,198 genes exceeded background in the monocyte transcriptome before and/or after treatment. Of these, 399 genes were significantly differentially modulated by EPO (p-value<0.05; 1.5% of total assessed genes; see table S3). Fold changes were low in this comparison (range 0.58–3.49); differential modulation of 211 genes was >1.10 or <0.90 fold. Strikingly, Euclidean clustering with these genes demonstrated that treated patient clustered close to his/her own baseline gene profile (figure 2 and figure S1). Although our intervention study was not designed to that purpose. clustering of patients did not seem to be associated with age, gender, or the presence of diabetes.

#### Specific gene expression changes

The 3 EPO injections only increased HBB and HBA2 expressions significantly with a fold change>1.35 when comparing group mean gene intensities. As mentioned, expression of these two genes may be attributed to reticulocyte contamination. Subsequently, to evaluate if short-term EPO treatment demonstrated monocyte gene modulations with regard to inflammation or oxidative stress we assessed gene expression changes in selected gene panels. None of the genes were differentially expressed with a p-value<0.05 (table S4).

#### Individual gene response to erythropoietin

Since the expression profile of the treated group did not substantially differ from baseline, we compared gene expression modulations in the twelve individual patients. The individual gene response to EPO was remarkably variable in all patients. Only two genes (HBB, HBA2) were induced and one gene downregulated (RAMP2) in more than two patients after treatment.

## Discussion

The present study is based on three of our recent studies in humans. The first showed altered leukocyte gene expression in untreated hypertensive patients, which was strongly attenuated in matched, well-treated patients [16]. In the other two papers, monocytes gene expressions in CKD [20] and end-stage renal disease [21] patients showed induction of the so-called suppressors of cytokine signaling, which modulate the Jak/Stat transcription pathway and steer the actions of IFNγ and IL6 [32]. Therefore, we hypothesized that monocytes, as cells involved in atherosclerosis [15], [33], could function as biosensors of the systemic environment of CRS patients with anemia and of the effects of short-term EPO treatment.

When comparing monocyte transcriptomes in CRS patients to healthy controls, one of the most remarkable observations is the limited number of changes, which could have a number of potential explanations. First, the study subjects were carefully selected, stable cardiorenal failure patients; the regular treatment, including angiotensin blockade (89%), statins (56%), acetylsalicylic acid (44%) and β-blockers (72%) may have dampened gene expression changes, even though the inflammatory environment was not completely normalized as judged from increased hsCRP levels. In this regard, monocytes displayed slightly higher numbers of genes with modulated expression in subjects with higher CRP values, than in subjects with lower CRP values. Unfortunately, the sample size is too small and the percentage of patients on medication too high to separate out the influence of any of the individual drugs. Second, it may be that the monocyte has adapted to the continuous exposure of stimuli, e.g. inflammation and oxidative stress. However, if this were the case, we would expect to see more ‘imprints’ of such adaptations in the transcriptome (e.g. more pronounced induction of anti-oxidant genes). Third, critical changes in monocytes of cardiorenal patients may be not achieved at the level of gene transcription but at the level of protein synthesis, modification and trafficking. It is unlikely that the choice of Illumina arrays underlies the paucity of changes, since this platform provided highly reproducible results and revealed important biological processes in monocytes [34]. In our own laboratory, the same platform yielded very strong transcriptional responses of endothelial cells in culture to IFNγ and IL6 [35]. Regarding the methodology applied in the present study, we did not correct for multiple testing using methods such as FDR, because of the small sample size. Therefore, the possibility exists that some of the reported changes in expression could be false positives. In another EPOCARES sub-study, which evaluated the responses of plasma hepcidin to the same short-term low dose EPO stimulation [24], we found clear responses in plasma hepcidin levels and reticulocyte numbers, clearly indicating that the dose of EPO was sufficiently strong to appreciably affect iron metabolism and erythropoiesis [23]. A last explanation is that the group is quite heterogeneous, as exemplified by the analysis of variation in log_2_ values of each patient.

The pathophysiology of CRS is complex and likely involves disruption of the pro-/anti-inflammatory and pro-/anti-oxidant balance, and enhanced activity of the SNS. We investigated whether monocytes reveal imprints of such alterations. Earlier studies in our group reported modulated expression of SOCS genes in monocytes of CKD patients [20]. We found increased CX3CR1 expression in CRS patients. CX3CR1 binds fractalkine (CX3CL1), a CX3C chemokine, which is expressed by activated endothelial cells and mediates adhesion and chemotaxis of CX3CR1 expressing monocytes and accumulation of macrophages in atherosclerotic lesions [36]. Transcription of some genes that stimulate inflammation (e.g. IL8, IL1RAP) was decreased in CRS patients compared to healthy controls, which may indicate negative feedback in response to inflammation. All-in-all, our analysis does not point to the cytokines IL6, IFNγ and TNFα, that have classically been associated with low grade inflammation in cardiovascular disease [37] and renal failure [38]. Nevertheless, the transcriptome seemed to be responsive to the level of inflammation, since higher CRP levels were associated with more changes in monocyte gene expression.

Considering the pro/anti-oxidant balance in CRS patients, expression changes were modest. Downregulation of genes encoding for proteins with anti-oxidative action is suggested (e.g. GPX3, PRDX3), though others were induced (DHRS9). Decreased expression of oxidative stress responsive gene DUSP1 and markers of early activation FOS and EGR1 possibly reflects downregulation of gene expression in activated cells from our patients. This phenomenon has been reported previously in patients with coronary artery disease [39].

The only change in CRS patients related to the SNS was increased expression of ADRB2, the adrenergic receptor β2 subtype that binds epinephrine and norepinephrine [40]. This gene is involved in coupling the SNS to immune cell function [41]. Sympathetic hyperactivity is present in patients with heart [42] and failure [43]. It was expected that chronic sympathetic hyperactivity would lead to downregulation of adrenoceptor density [44], which underlies the decrease in β-adrenoreceptor-mediated responsiveness characteristic [45], [46]. However, β-blockade was previously shown to increase adrenoreceptor density in leukocytes. Patients with the highest catecholamine levels had the greatest rise in receptor density following β-blockade [44]. Albeit speculative, since 72% of our patients received β-blockade, higher ADRB2 expression levels may indicate higher baseline sympathetic activity compared to our healthy controls.

Our second aim was to evaluate short-term treatment with a currently recommended EPO dose for renal anemia in this patient population. In another arm of this study, there was a clear decrease in hepcidin, an increase in reticulocytes, an increase in serum transferrin receptor and in iron saturation [24] after 2 weeks of EPO. At 26 weeks, all of the patients except for 2 had a clear increase in haemoglobin levels [47], and/or had been subjected to phlebotomy (this is in the group in which in the longer run, Hgb levels were kept stable by phlebotomy). We postulated previously that EPO may dampen activated inflammatory and oxidative stress systems in CRS patients [5]. By evaluating transcriptomes after 18 days of EPO therapy, direct effects of EPO should be discriminated from hematopoietic effects. However, EPO treatment did not substantially modulate the monocyte transcriptome in our study. The transcriptome after short-term EPO therapy closely clustered with to the baseline expression profile for every patient in our cluster analysis. This suggests that individual differences overrule the direct, short-term effects of EPO. Furthermore, we found a highly variable gene expression response to EPO in all patients, which hampers the detection of group differences. The finding that EPO response at gene expression level is so diverse may point at the variable clinical response to EPO.

Several reasons may underlie the unexpected lack in differential gene expression in response to EPO treatment. First, it was remarkable that individual responses to EPO were so variable, which hinders detection of global gene expression changes between groups. Despite careful selection of patients and optimally standardizing medical treatment, individual variations still seem to dominate the effect of EPO therapy. Microarray technology has shown to reflect the clinical response to medical therapy [16], [17]. Our study certainly does not rule out that transcriptome analysis on circulating cells could be applied to monitor early responses to EPO, however, another cell type may better reflect the changes induces by EPO [15]. One could think of endothelial progenitor cells, that might better reflect the response of a target tissue to EPO, or reticulocytes, that might better reflect the response of the bone marrow to EPO. Studying targets organ cells per se obviously is more invasive. Second, the dose and duration of EPO treatment must be considered. Various animal studies have shown protective effects of EPO in acute ischemia/reperfusion injury of the heart and kidney [13], [14]. EPO doses used in these studies are considerably higher, ranging from 3000–5000 U/kg, which is much higher than the dosages usually applied in CKD and end-stage renal disease patients. However, recent evidence from the CHOIR study shows that chronic administration of high EPO dose (mean dose 11215 IU/wk) is associated with adverse clinical events. Since we treat our patients up to 1 year, we chose a currently recommended dose for the treatment of renal anemia of 50 IU/kg/wk (i.e. approximately 3000 IU/wk), and not a short-term high dose treatment. As alternative to the present design, that aimed to study both short and long term effects of EPO which did not justify using a higher dose, one could possibly safely study the effect of one single dose of a higher concentration of EPO in humans. Altogether, the present data do not support a beneficial role for non-hematopoietic, short-term effects of low dose EPO. More importantly, using monocyte transcriptomes, we were unable to demonstrate any harmful effects.

In summary, we demonstrate that differences in the gene expression of monocytes, being biosensors of the pro-atherogenic environment and mediators of early atherosclerosis, are limited in our study subjects. Nevertheless, the observed changes point at two of the systems we have proposed as important connectors in combined heart and renal failure, being inflammation and oxidative stress. We also demonstrate that in monocytes, response in gene expression to short-term administration of the currently recommended dose of EPO is very limited, indicating that a dose that is commonly used to combat the EPO deficiency of renal anemia does not exert important non-hematopoietic effects on this pivotal cell. Fortunately, we also have been unable to identify any undesirable effects of this widely applied dose of EPO. Further studies are necessary to investigate whether other immune cells involved in the inflammatory response and in atherosclerosis may be more sensitive to this recommended dose of EPO and whether higher doses of EPO as used in cardiovascular trials do affect monocyte gene expression.

## Supporting Information

Figure S1 Euclidean cluster analysis for patients before and after erythropoietin treatment. Patient number and time point of sample collection (patient 1, timepoint 1 = 11; patient 1, timepoint 2 = 12, etc) are listed in order of monocyte transcriptomes similarity. The closer samples are depicted to each other, the more comparable transcriptomes are.
(TIF)

Table S1 Monocyte gene expression differences in CRS patients at baseline (n = 18) compared to healthy controls (n = 12).
(PDF)

Table S2 Cardiorenal connector monocyte gene expression panel: differences in CRS patients at baseline (n = 18) compared to healthy controls (n = 12).
(PDF)

Table S3 Monocyte gene expression changes in CRS patients (n = 12) after 2 weeks of EPO treatment.
(PDF)

Table S4 Cardiorenal connector monocyte gene expression panel: changes in CRS patients (n = 12) after 2 weeks of EPO treatment.
(PDF)

## References

[1] Hillege HL, Nitsch D, Pfeffer MA, Swedberg K, McMurray JJ, et al (2006) Renal function as a predictor of outcome in a broad spectrum of patients with heart failure. Circulation 113: 671–678. 16461840 10.1161/CIRCULATIONAHA.105.580506

[2] Foley RN, Parfrey PS, Sarnak MJ (1998) Clinical epidemiology of cardiovascular disease in chronic renal disease. Am J Kidney Dis 32: S112–119. 9820470 10.1053/ajkd.1998.v32.pm9820470

[3] Bongartz LG, Cramer MJ, Doevendans PA, Joles JA, Braam B (2005) The severe cardiorenal syndrome: ‘Guyton revisited’. Eur Heart J 26: 11–17. 15615794 10.1093/eurheartj/ehi020

[4] Coulon S, Dussiot M, Grapton D, Maciel TT, Wang PH, et al (2011) Polymeric IgA1 controls erythroblast proliferation and accelerates erythropoiesis recovery in anemia. Nature medicine 17: 1456–1465. 10.1038/nm.2462 22019886

[5] Jie KE, Verhaar MC, Cramer MJ, van der Putten K, Gaillard CA, et al (2006) Erythropoietin and the cardiorenal syndrome: cellular mechanisms on the cardiorenal connectors. Am J Physiol Renal Physiol 291: F932–944. 16885153 10.1152/ajprenal.00200.2006

[6] Lisowska KA, Debska-Slizien A, Bryl E, Rutkowski B, Witkowski JM (2010) Erythropoietin receptor is expressed on human peripheral blood T and B lymphocytes and monocytes and is modulated by recombinant human erythropoietin treatment. Artificial organs 34: 654–662. 20528849 10.1111/j.1525-1594.2009.00948.x

[7] Groenveld HF, Januzzi JL, Damman K, van Wijngaarden J, Hillege HL, et al (2008) Anemia and mortality in heart failure patients a systematic review and meta-analysis. J Am Coll Cardiol 52: 818–827. 18755344 10.1016/j.jacc.2008.04.061

[8] Sarnak MJ, Tighiouart H, Manjunath G, MacLeod B, Griffith J, et al (2002) Anemia as a risk factor for cardiovascular disease in The Atherosclerosis Risk in Communities (ARIC) study. J Am Coll Cardiol 40: 27–33. 12103252 10.1016/s0735-1097(02)01938-1

[9] Drueke TB, Locatelli F, Clyne N, Eckardt KU, Macdougall IC, et al (2006) Normalization of hemoglobin level in patients with chronic kidney disease and anemia. N Engl J Med 355: 2071–2084. 17108342 10.1056/NEJMoa062276

[10] Pfeffer MA, Burdmann EA, Chen YJ, Cooper ME, De Zeeuw D, et al (2009) A trial of darbepoietin alpha in type 2 diabetes and chronic kidney disease. N Engl J Med 361: 2019–2032. 19880844 10.1056/NEJMoa0907845

[11] Singh AK, Szczech L, Tang KL, Barnhart H, Sapp S, et al (2006) Correction of anemia with epoetin alfa in chronic kidney disease. N Engl J Med 355: 2085–2098. 17108343 10.1056/NEJMoa065485

[12] Szczech LA, Barnhart HX, Inrig JK, Reddan DN, Sapp S, et al (2008) Secondary analysis of the CHOIR trial epoetin-alpha dose and achieved hemoglobin outcomes. Kidney Int 74: 791–798. 18596733 10.1038/ki.2008.295 PMC2902279

[13] Chang YK, Choi DE, Na KR, Lee SJ, Suh KS, et al (2009) Erythropoietin attenuates renal injury in an experimental model of rat unilateral ureteral obstruction via anti-inflammatory and anti-apoptotic effects. J Urol 181: 1434–1443. 19157461 10.1016/j.juro.2008.10.105

[14] Parsa CJ, Matsumoto A, Kim J, Riel RU, Pascal LS, et al (2003) A novel protective effect of erythropoietin in the infarcted heart. J Clin Invest 112: 999–1007. 14523037 10.1172/JCI18200 PMC198525

[15] Ardigo D, Gaillard CA, Braam B (2007) Application of leukocyte transcriptomes to assess systemic consequences of risk factors for cardiovascular disease. Clin Chem Lab Med 45: 1109–1120. 17635069 10.1515/CCLM.2007.261

[16] Chon H, Gaillard CA, van der Meijden BB, Dijstelbloem HM, Kraaijenhagen RJ, et al (2004) Broadly altered gene expression in blood leukocytes in essential hypertension is absent during treatment. Hypertension 43: 947–951. 15007037 10.1161/01.HYP.0000123071.35142.72

[17] Wibaut-Berlaimont V, Randi AM, Mandryko V, Lunnon MW, Haskard DO, et al (2005) Atorvastatin affects leukocyte gene expression in dyslipidemia patients: in vivo regulation of hemostasis, inflammation and apoptosis. J Thromb Haemost 3: 677–685. 15842352 10.1111/j.1538-7836.2005.01211.x

[18] AbdAlla S, Lother H, Langer A, el Faramawy Y, Quitterer U (2004) Factor XIIIA transglutaminase crosslinks AT1 receptor dimers of monocytes at the onset of atherosclerosis. Cell 119: 343–354. 15507206 10.1016/j.cell.2004.10.006

[19] Sprague AH, Khalil RA (2009) Inflammatory cytokines in vascular dysfunction and vascular disease. Biochem Pharmacol 10.1016/j.bcp.2009.04.029 PMC2730638 19413999

[20] Rastmanesh MM, Bluyssen HA, Joles JA, Boer P, Willekes N, et al (2008) Increased expression of SOCS3 in monocytes and SOCS1 in lymphocytes correlates with progressive loss of renal function and cardiovascular risk factors in chronic kidney disease. Eur J Pharmacol 593: 99–104. 18656467 10.1016/j.ejphar.2008.07.013

[21] Rastmanesh MM, Braam B, Joles JA, Boer P, Bluyssen HA (2009) Increased SOCS expression in peripheral blood mononuclear cells of end stage renal disease patients is related to inflammation and dialysis modality. Eur J Pharmacol 602: 163–167. 19041303 10.1016/j.ejphar.2008.11.014

[22] Meier P, Antonov J, Zbinden R, Kuhn A, Zbinden S, et al (2009) Non-invasive gene-expression-based detection of well-developed collateral function in individuals with and without coronary artery disease. Heart 95: 900–908. 18728069 10.1136/hrt.2008.145383

[23] van der Putten K, Jie KE, Emans ME, Verhaar MC, Joles JA, et al (Prelim citation) Erythropoietin treatment in patients with combined heart and renal failure: objectives and design of the EPOCARES study. J Nephrol 23: 363–368. 20383871

[24] van der Putten K, Jie KE, van den Broek D, Kraaijenhagen RJ, Laarakkers C, et al (2010) Hepcidin-25 is a marker of the response rather than resistance to exogenous erythropoietin in chronic kidney disease/chronic heart failure patients. Eur J Heart Fail 12: 943–950. 20601671 10.1093/eurjhf/hfq099

[25] Dickstein K, Cohen-Solal A, Filippatos G, McMurray JJ, Ponikowski P, et al (2008) ESC Guidelines for the diagnosis and treatment of acute and chronic heart failure 2008: the Task Force for the Diagnosis and Treatment of Acute and Chronic Heart Failure 2008 of the European Society of Cardiology. Developed in collaboration with the Heart Failure Association of the ESC (HFA) and endorsed by the European Society of Intensive Care Medicine (ESICM). Eur Heart J 29: 2388–2442. 18799522 10.1093/eurheartj/ehn309

[26] Paulus WJ, Tschope C, Sanderson JE, Rusconi C, Flachskampf FA, et al (2007) How to diagnose diastolic heart failure: a consensus statement on the diagnosis of heart failure with normal left ventricular ejection fraction by the Heart Failure and Echocardiography Associations of the European Society of Cardiology. Eur Heart J 28: 2539–2550. 17428822 10.1093/eurheartj/ehm037

[27] Pfeffer MA, Burdmann EA, Chen CY, Cooper ME, de Zeeuw D, et al (2009) A trial of darbepoetin alfa in type 2 diabetes and chronic kidney disease. N Engl J Med 361: 2019–2032. 19880844 10.1056/NEJMoa0907845

[28] Blazejczyk M, Miron M, Nadon R (2007) FlexArray: A statistical data analysis software for gene expression microarrays. Montreal, Quebec, Canada.: Genome Quebec, Canada.

[29] Baldi P, Long AD (2001) A Bayesian framework for the analysis of microarray expression data: regularized t -test and statistical inferences of gene changes. Bioinformatics 17: 509–519. 11395427 10.1093/bioinformatics/17.6.509

[30] Saeed AI, Bhagabati NK, Braisted JC, Liang W, Sharov V, et al (2006) TM4 microarray software suite. Methods in enzymology 411: 134–193. 16939790 10.1016/S0076-6879(06)11009-5

[31] Edgar R, Domrachev M, Lash AE (2002) Gene Expression Omnibus: NCBI gene expression and hybridization array data repository. Nucleic Acids Res 30: 207–210. 11752295 10.1093/nar/30.1.207 PMC99122

[32] Lang R, Pauleau AL, Parganas E, Takahashi Y, Mages J, et al (2003) SOCS3 regulates the plasticity of gp130 signaling. Nat Immunol 4: 546–550. 12754506 10.1038/ni932

[33] Chon H, Verhaar MC, Koomans HA, Joles JA, Braam B (2006) Role of circulating karyocytes in the initiation and progression of atherosclerosis. Hypertension 47: 803–810. 16520401 10.1161/01.HYP.0000210554.61293.90

[34] Maouche S, Poirier O, Godefroy T, Olaso R, Gut I, et al (2008) Performance comparison of two microarray platforms to assess differential gene expression in human monocyte and macrophage cells. BMC Genomics 9: 302. 18578872 10.1186/1471-2164-9-302 PMC2464609

[35] Bluyssen HA, Rastmanesh MM, Tilburgs C, Jie K, Wesseling S, et al (2010) IFN gamma-dependent SOCS3 expression inhibits IL-6-induced STAT3 phosphorylation and differentially affects IL-6 mediated transcriptional responses in endothelial cells. Am J Physiol Cell Physiol 299: C354–362. 20484656 10.1152/ajpcell.00513.2009

[36] Combadiere C, Potteaux S, Gao JL, Esposito B, Casanova S, et al (2003) Decreased atherosclerotic lesion formation in CX3CR1/apolipoprotein E double knockout mice. Circulation 107: 1009–1016. 12600915 10.1161/01.cir.0000057548.68243.42

[37] Lind L (2003) Circulating markers of inflammation and atherosclerosis. Atherosclerosis 169: 203–214. 12921971 10.1016/s0021-9150(03)00012-1

[38] Malaponte G, Bevelacqua V, Fatuzzo P, Rapisarda F, Emmanuele G, et al (2002) IL-1beta, TNF-alpha and IL-6 release from monocytes in haemodialysis patients in relation to dialytic age. Nephrol Dial Transplant 17: 1964–1970. 12401854 10.1093/ndt/17.11.1964

[39] Schirmer SH, Fledderus JO, van der Laan AM, van der Pouw-Kraan TC, Moerland PD, et al (2009) Suppression of inflammatory signaling in monocytes from patients with coronary artery disease. J Mol Cell Cardiol 46: 177–185. 19059264 10.1016/j.yjmcc.2008.10.029

[40] Strader CD, Fong TM, Tota MR, Underwood D, Dixon RA (1994) Structure and function of G protein-coupled receptors. Annu Rev Biochem 63: 101–132. 7979235 10.1146/annurev.bi.63.070194.000533

[41] Elenkov IJ, Wilder RL, Chrousos GP, Vizi ES (2000) The sympathetic nerve–an integrative interface between two supersystems: the brain and the immune system. Pharmacol Rev 52: 595–638. 11121511

[42] Jackson G, Gibbs CR, Davies MK, Lip GY (2000) ABC of heart failure. Pathophysiology. BMJ 320: 167–170. 10634740 10.1136/bmj.320.7228.167 PMC1128747

[43] Koomans HA, Blankestijn PJ, Joles JA (2004) Sympathetic hyperactivity in chronic renal failure: a wake-up call. J Am Soc Nephrol 15: 524–537. 14978154 10.1097/01.asn.0000113320.57127.b9

[44] Fraser J, Nadeau J, Robertson D, Wood AJ (1981) Regulation of human leukocyte beta receptors by endogenous catecholamines: relationship of leukocyte beta receptor density to the cardiac sensitivity to isoproterenol. J Clin Invest 67: 1777–1784. 6263952 10.1172/JCI110217 PMC370756

[45] Bristow MR, Ginsburg R, Minobe W, Cubicciotti RS, Sageman WS, et al (1982) Decreased catecholamine sensitivity and beta-adrenergic-receptor density in failing human hearts. N Engl J Med 307: 205–211. 6283349 10.1056/NEJM198207223070401

[46] Brodde OE, Daul A (1986) Impaired regulation of alpha- and beta-adrenoceptor function in chronic renal insufficiency. Contrib Nephrol 50: 28–35. 3026728

[47] Jie KE, van der Putten K, Bergevoet MW, Doevendans PA, Gaillard CA, et al (2011) Short- and long-term effects of erythropoietin treatment on endothelial progenitor cell levels in patients with cardiorenal syndrome. Heart 97: 60–65. 21071558 10.1136/hrt.2010.194654 PMC3002834

