# Supplementary figures and images for: Short-Term Erythropoietin Treatment Does Not Substantially Modulate Monocyte Transcriptomes of Patients with Combined Heart and Renal Failure
Source: PLoS One. 2012 Sep 5;7(9):e41339. doi: 10.1371/journal.pone.0041339 (PMC3434212; doi:10.1371/journal.pone.0041339)

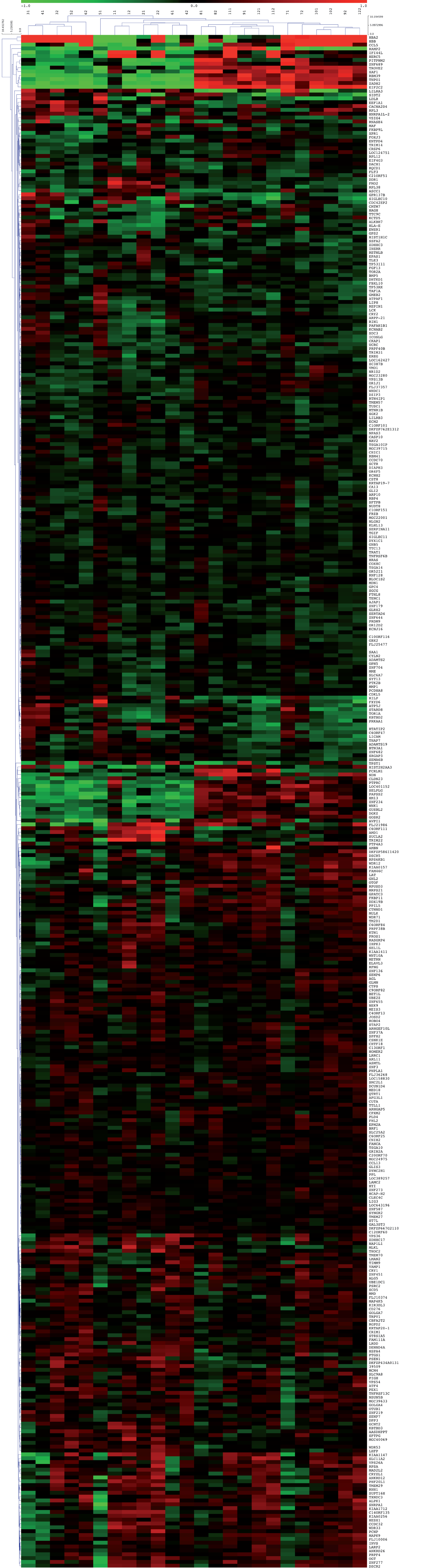

Supplement: Figure S1 — Euclidean cluster analysis for patients before and after erythropoietin treatment. Patient number and time point of sample collection (patient 1, timepoint 1 = 11; patient 1, timepoint 2 = 12, etc) are listed in order of monocyte transcriptomes similarity. The closer samples are depicted to each other, the more comparable transcriptomes are. (TIF) [file pone.0041339.s001.tif]
