# Supplementary material for: Short-Term Erythropoietin Treatment Does Not Substantially Modulate Monocyte Transcriptomes of Patients with Combined Heart and Renal Failure
Source: PLoS One. 2012 Sep 5;7(9):e41339. doi: 10.1371/journal.pone.0041339 (PMC3434212; doi:10.1371/journal.pone.0041339)
Supplement: Table S1 — Monocyte gene expression differences in CRS patients at baseline (n = 18) compared to healthy controls (n = 12). (PDF) [file pone.0041339.s002.pdf]

Supplementary material table 1: Monocyte gene expression differences in CRS patients at baseline (n=18) compared to healthy controls (n=12)

Transcript	Symbol	Definition	Healthy controls log mean intensity	Patients at baseline log mean intensity	Fold change	P-value
**UPREGULATED**						
NM_018487.2	HCA112	Homo sapiens transmembrane protein 176A (TMEM176A), mRNA.	8.33	9.09	1.70	0.0373
NM_012456.1	TIMM10	Homo sapiens translocase of inner mitochondrial membrane 10 homolog (yeast) (TIMM10), mRNA.	7.70	8.37	1.59	0.0000
NM_017911.1	C22ORF8	Homo sapiens family with sequence similarity 118, member A (FAM118A), mRNA.	6.89	7.55	1.58	0.0118
NM_001337.3	CX3CR1	Homo sapiens chemokine (C-X3-C motif) receptor 1 (CX3CR1), mRNA.	10.86	11.43	1.49	0.0002
NM_006498.2	LGALS2	Homo sapiens lectin, galactoside-binding, soluble, 2 (LGALS2), mRNA.	8.20	8.75	1.46	0.0284
NM_000024.3	ADRB2	Homo sapiens adrenergic, beta-2-, receptor, surface (ADRB2), mRNA.	8.47	9.00	1.45	0.0015
NM_005771.3	DHRS9	Homo sapiens dehydrogenase/reductase (SDR family) member 9 (DHRS9), transcript variant 1, mRNA.	8.38	8.90	1.43	0.0080
NM_198097.1	C7ORF28B	Homo sapiens chromosome 7 open reading frame 28B (C7orf28B), mRNA.	8.40	8.92	1.43	0.0053
NM_001343.1	DAB2	Homo sapiens disabled homolog 2, mitogen-responsive phosphoprotein (Drosophila) (DAB2), mRNA.	6.92	7.44	1.43	0.0003
NM_016021.2	UBE2J1	Homo sapiens ubiquitin-conjugating enzyme E2, J1 (UBC6 homolog, yeast) (UBE2J1), mRNA.	8.42	8.93	1.42	0.0006
NM_030670.1	PTPRO	Homo sapiens protein tyrosine phosphatase, receptor type, O (PTPRO), transcript variant 6, mRNA.	6.91	7.38	1.39	0.0000
NM_001008566.1	TPST2	Homo sapiens tyrosylprotein sulfotransferase 2 (TPST2), transcript variant 1, mRNA.	9.73	10.20	1.38	0.0014
NM_030671.1	PTPRO	Homo sapiens protein tyrosine phosphatase, receptor type, O (PTPRO), transcript variant 5, mRNA.	7.37	7.82	1.37	0.0006
NR_003038.1	SNHG5	Homo sapiens small nucleolar RNA host gene (non-protein coding) 5 (SNHG5) on chromosome 6.	10.13	10.58	1.36	0.0365
NM_080914.1	ASGR2	Homo sapiens asialoglycoprotein receptor 2 (ASGR2), transcript variant 3, mRNA.	9.44	9.88	1.36	0.0029
NM_016613.4	C4ORF18	Homo sapiens chromosome 4 open reading frame 18 (C4orf18), transcript variant 2, mRNA.	8.93	9.36	1.35	0.0158
NM_016613.4	C4ORF18	Homo sapiens chromosome 4 open reading frame 18 (C4orf18), transcript variant 2, mRNA.	9.57	10.00	1.35	0.0033
NM_152594.1	SPRED1	Homo sapiens sprouty-related, EVH1 domain containing 1 (SPRED1), mRNA.	8.11	8.54	1.34	0.0040
NM_005771.3	DHRS9	Homo sapiens dehydrogenase/reductase (SDR family) member 9 (DHRS9), transcript variant 1, mRNA.	7.13	7.54	1.33	0.0057
NM_144580.1	C1ORF85	Homo sapiens chromosome 1 open reading frame 85 (C1orf85), mRNA.	9.54	9.94	1.32	0.0099
NM_021244.2	RRAGD	Homo sapiens Ras-related GTP binding D (RRAGD), mRNA.	7.85	8.24	1.31	0.0137
NM_004383.1	CSK	Homo sapiens c-src tyrosine kinase (CSK), mRNA.	10.59	10.98	1.31	0.0001
NM_004949.2	DSC2	Homo sapiens desmocollin 2 (DSC2), transcript variant Dsc2b, mRNA.	7.42	7.81	1.31	0.0207
NM_031890.2	CECR6	Homo sapiens cat eye syndrome chromosome region, candidate 6 (CECR6), mRNA.	7.75	8.14	1.30	0.0003
NM_080914.1	ASGR2	Homo sapiens asialoglycoprotein receptor 2 (ASGR2), transcript variant 3, mRNA.	9.32	9.70	1.30	0.0167
NM_000129.2	F13A1	Homo sapiens coagulation factor XIII, A1 polypeptide (F13A1), mRNA.	8.85	9.21	1.29	0.0127
NM_003494.2	DYSF	Homo sapiens dysferlin, limb girdle muscular dystrophy 2B (autosomal recessive) (DYSF), mRNA.	8.67	9.03	1.28	0.0064
NM_013385.2	PSCD4	Homo sapiens pleckstrin homology, Sec7 and coiled-coil domains 4 (PSCD4), mRNA.	11.30	11.65	1.27	0.0015
NM_002935.2	RNASE3	Homo sapiens ribonuclease, RNase A family, 3 (eosinophil cationic protein) (RNASE3), mRNA.	6.72	7.07	1.27	0.0093
NM_004462.3	FDFT1	Homo sapiens farnesyl-diphosphate farnesyltransferase 1 (FDFT1), mRNA.	9.74	10.08	1.27	0.0014
NM_198595.1	AFAP	Homo sapiens actin filament associated protein (AFAP), transcript variant 2, mRNA.	6.46	6.79	1.26	0.0034
NM_020223.1	FAM20C	Homo sapiens family with sequence similarity 20, member C (FAM20C), mRNA.	7.73	8.07	1.26	0.0017
NM_018476.3	BEX1	Homo sapiens brain expressed, X-linked 1 (BEX1), mRNA.	6.48	6.81	1.26	0.0089
NM_182616.1	C15ORF38	Homo sapiens chromosome 15 open reading frame 38 (C15orf38), mRNA.	7.45	7.78	1.26	0.0211
NM_013451.2	FER1L3	Homo sapiens fer-1-like 3, myoferlin (C. elegans) (FER1L3), transcript variant 1, mRNA.	8.84	9.17	1.26	0.0030
NM_005902.3	SMAD3	Homo sapiens SMAD family member 3 (SMAD3), mRNA.	7.78	8.11	1.25	0.0005
NM_000820.1	GAS6	Homo sapiens growth arrest-specific 6 (GAS6), mRNA.	6.86	7.19	1.25	0.0013
NM_003516.2	HIST2H2AA3	Homo sapiens histone cluster 2, H2aa3 (HIST2H2AA3), mRNA.	9.07	9.40	1.25	0.0156
XM_942084.1	GAS6	PREDICTED: Homo sapiens growth arrest-specific 6, transcript variant 2 (GAS6), mRNA.	7.17	7.49	1.25	0.0025
NM_002934.2	RNASE2	Homo sapiens ribonuclease, RNase A family, 2 (liver, eosinophil-derived neurotoxin) (RNASE2), mRNA.	10.51	10.83	1.24	0.0461
NM_000491.2	C1QB	Homo sapiens complement component 1, q subcomponent, beta polypeptide (C1QB), mRNA.	6.87	7.19	1.24	0.0344
NM_080759.3	DACH1	Homo sapiens dachshund homolog 1 (Drosophila) (DACH1), transcript variant 1, mRNA.	6.83	7.13	1.24	0.0010
NM_006270.2	RRAS	Homo sapiens related RAS viral (r-ras) oncogene homolog (RRAS), mRNA.	9.30	9.61	1.23	0.0064
NM_004364.2	CEBPA	Homo sapiens CCAAT/enhancer binding protein (C/EBP), alpha (CEBPA), mRNA.	9.56	9.86	1.23	0.0066
NM_005817.3	M6PRBP1	Homo sapiens mannose-6-phosphate receptor binding protein 1 (M6PRBP1), mRNA.	10.66	10.95	1.23	0.0030
NM_173078.2	SLITRK4	Homo sapiens SLIT and NTRK-like family, member 4 (SLITRK4), mRNA.	7.87	8.17	1.23	0.0065
NM_021965.3	PGM5	Homo sapiens phosphoglucomutase 5 (PGM5), mRNA.	6.79	7.08	1.23	0.0430
NM_000355.2	TCN2	Homo sapiens transcobalamin II; macrocytic anemia (TCN2), mRNA.	7.30	7.59	1.23	0.0129
NM_006745.3	SC4MOL	Homo sapiens sterol-C4-methyl oxidase-like (SC4MOL), transcript variant 1, mRNA.	7.13	7.42	1.22	0.0024
NM_001004340.1	FCGR1B	Homo sapiens Fc fragment of IgG, high affinity Ib, receptor (CD64) (FCGR1B), transcript variant 2, mRNA.	8.33	8.62	1.22	0.0488
NM_001006932.1	RPS6KA2	Homo sapiens ribosomal protein S6 kinase, 90kDa, polypeptide 2 (RPS6KA2), transcript variant 2, mRNA.	6.45	6.74	1.22	0.0346
NM_080593.1	HIST1H2BK	Homo sapiens histone cluster 1, H2bk (HIST1H2BK), mRNA.	9.78	10.07	1.22	0.0153
NM_001008485.1	SLC41A3	Homo sapiens solute carrier family 41, member 3 (SLC41A3), transcript variant 1, mRNA.	8.03	8.32	1.22	0.0200
NM_004843.2	IL27RA	Homo sapiens interleukin 27 receptor, alpha (IL27RA), mRNA.	8.79	9.08	1.22	0.0012
NM_173511.2	ALS2CR13	Homo sapiens amyotrophic lateral sclerosis 2 (juvenile) chromosome region, candidate 13 (ALS2CR13), mRNA.	9.75	10.03	1.21	0.0382
NM_005885.2	MARCH6	Homo sapiens membrane-associated ring finger (C3HC4) 6 (MARCH6), mRNA.	7.45	7.73	1.21	0.0244
NM_001425.1	EMP3	Homo sapiens epithelial membrane protein 3 (EMP3), mRNA.	12.05	12.32	1.21	0.0051
NM_014584.1	ERO1L	Homo sapiens ERO1-like (S. cerevisiae) (ERO1L), mRNA.	7.65	7.92	1.21	0.0022
NM_000270.1	NP	Homo sapiens nucleoside phosphorylase (NP), mRNA.	9.27	9.55	1.21	0.0254
NM_004672.3	MAP3K6	Homo sapiens mitogen-activated protein kinase kinase kinase 6 (MAP3K6), mRNA.	8.30	8.57	1.21	0.0180
NM_002209.1	ITGAL	Homo sapiens integrin, alpha L (antigen CD11A (p180), lymphocyte function-associated antigen 1; alpha polypeptide) (ITGAL), mRNA.	10.19	10.46	1.21	0.0047
NM_080792.1	PTPNS1	Homo sapiens protein tyrosine phosphatase, non-receptor type substrate 1 (PTPNS1), mRNA.	11.70	11.97	1.21	0.0144
NM_013337.2	TIMM22	Homo sapiens translocase of inner mitochondrial membrane 22 homolog (yeast) (TIMM22), mRNA.	8.39	8.66	1.21	0.0284

NM_152773.2	MGC33212	Homo sapiens hypothetical protein MGC33212 (MGC33212), mRNA.	7.04	7.31	1.20	0.0106
NM_153280.1	UBE1	Homo sapiens ubiquitin-activating enzyme E1 (UBE1), transcript variant 2, mRNA.	9.08	9.35	1.20	0.0083
NM_004099.4	STOM	Homo sapiens stomatin (STOM), transcript variant 1, mRNA.	8.74	9.01	1.20	0.0292
NM_005828.2	WDR68	Homo sapiens WD repeat domain 68 (WDR68), transcript variant 1, mRNA.	9.11	9.37	1.20	0.0142
NM_004090.2	DUSP3	Homo sapiens dual specificity phosphatase 3 (vaccinia virus phosphatase VH1-related) (DUSP3), mRNA.	9.69	9.96	1.20	0.0498
NM_000110.2	DPYD	Homo sapiens dihydropyrimidine dehydrogenase (DPYD), mRNA.	8.66	8.93	1.20	0.0257
NM_000067.1	CA2	Homo sapiens carbonic anhydrase II (CA2), mRNA.	7.09	7.35	1.20	0.0058
NM_016562.3	TLR7	Homo sapiens toll-like receptor 7 (TLR7), mRNA.	9.03	9.30	1.20	0.0403
NM_006942.1	SOX15	Homo sapiens SRY (sex determining region Y)-box 15 (SOX15), mRNA.	6.72	6.97	1.20	0.0120
NM_032350.3	MGC11257	Homo sapiens hypothetical protein MGC11257 (MGC11257), mRNA.	9.47	9.73	1.20	0.0283
NM_001386.4	DPYSL2	Homo sapiens dihydropyrimidinase-like 2 (DPYSL2), mRNA.	11.47	11.73	1.20	0.0320
NM_014339.3	IL17R	Homo sapiens interleukin 17 receptor (IL17R), mRNA.	8.26	8.52	1.19	0.0117
NM_021708.1	LAIR1	Homo sapiens leukocyte-associated Ig-like receptor 1 (LAIR1), transcript variant c, mRNA.	7.25	7.50	1.19	0.0370
NM_182827.1	FKBP9L	Homo sapiens FK506 binding protein 9-like (FKBP9L), mRNA.	6.74	6.99	1.19	0.0017
NM_001014763.1	ETFB	Homo sapiens electron-transfer-flavoprotein, beta polypeptide (ETFB), transcript variant 2, mRNA.	9.73	9.98	1.19	0.0259
NM_015179.2	KIAA0690	Homo sapiens ribosomal RNA processing 12 homolog (S. cerevisiae) (RRP12), mRNA.	8.13	8.38	1.19	0.0418
NM_005319.3	HIST1H1C	Homo sapiens histone cluster 1, H1c (HIST1H1C), mRNA.	6.80	7.05	1.19	0.0027
NM_003129.3	SQLE	Homo sapiens squalene epoxidase (SQLE), mRNA.	6.93	7.17	1.18	0.0134
NM_138452.1	DHRS1	Homo sapiens dehydrogenase/reductase (SDR family) member 1 (DHRS1), mRNA.	8.82	9.06	1.18	0.0087
NM_001002235.1	SERPINA1	Homo sapiens serpin peptidase inhibitor, clade A (alpha-1 antiproteinase, antitrypsin), member 1 (SERPINA1), transcript variant 3, mRNA.	12.02	12.26	1.18	0.0426
NM_018440.3	PAG1	Homo sapiens phosphoprotein associated with glycosphingolipid microdomains 1 (PAG1), mRNA.	8.16	8.40	1.18	0.0299
NM_133171.2	ELMO2	Homo sapiens engulfment and cell motility 2 (ELMO2), transcript variant 1, mRNA.	8.64	8.88	1.18	0.0366
NM_000391.2	TPP1	Homo sapiens tripeptidyl peptidase I (TPP1), mRNA.	10.76	10.99	1.18	0.0169
NM_001009566.1	CLSTN1	Homo sapiens calsyntenin 1 (CLSTN1), transcript variant 1, mRNA.	8.61	8.85	1.18	0.0094
NM_006367.2	CAP1	Homo sapiens CAP, adenylate cyclase-associated protein 1 (yeast) (CAP1), mRNA.	12.37	12.60	1.18	0.0318
NM_014449.1	GPR162	Homo sapiens G protein-coupled receptor 162 (GPR162), transcript variant A-1, mRNA.	10.37	10.60	1.17	0.0318
NM_013410.2	AK3L1	Homo sapiens adenylate kinase 3-like 1 (AK3L1), nuclear gene encoding mitochondrial protein, transcript variant 6, mRNA.	6.95	7.18	1.17	0.0026
NM_025201.3	PLEKHQ1	Homo sapiens pleckstrin homology domain containing, family Q member 1 (PLEKHQ1), mRNA.	8.55	8.78	1.17	0.0473
NM_004740.3	TIAF1	Homo sapiens TGFB1-induced anti-apoptotic factor 1 (TIAF1), mRNA.	9.41	9.64	1.17	0.0237
NM_014877.2	HELZ	Homo sapiens helicase with zinc finger (HELZ), mRNA.	8.34	8.57	1.17	0.0125
NM_002468.2	MYD88	Homo sapiens myeloid differentiation primary response gene (88) (MYD88), mRNA.	10.10	10.33	1.17	0.0449
NM_018653.3	GPRC5C	Homo sapiens G protein-coupled receptor, family C, group 5, member C (GPRC5C), transcript variant 2, mRNA.	6.49	6.71	1.17	0.0073
NM_198565.1	LRRC33	Homo sapiens leucine rich repeat containing 33 (LRRC33), mRNA.	8.80	9.02	1.17	0.0268
NM_002562.4	P2RX7	Homo sapiens purinergic receptor P2X, ligand-gated ion channel, 7 (P2RX7), transcript variant 1, mRNA.	9.07	9.29	1.17	0.0255
NM_170600.1	SH2D3C	Homo sapiens SH2 domain containing 3C (SH2D3C), transcript variant 2, mRNA.	8.95	9.17	1.16	0.0254
NM_001008213.1	OPTN	Homo sapiens optineurin (OPTN), transcript variant 4, mRNA.	6.61	6.82	1.16	0.0046
NM_080549.2	PTPN6	Homo sapiens protein tyrosine phosphatase, non-receptor type 6 (PTPN6), transcript variant 3, mRNA.	10.65	10.87	1.16	0.0238
NM_138570.1	MGC15523	Homo sapiens hypothetical protein MGC15523 (MGC15523), mRNA.	7.19	7.41	1.16	0.0404
NM_024321.3	MGC10433	Homo sapiens hypothetical protein MGC10433 (MGC10433), mRNA.	8.09	8.31	1.16	0.0213
NM_020148.1	SPIRE1	Homo sapiens spire homolog 1 (Drosophila) (SPIRE1), mRNA.	7.14	7.36	1.16	0.0416
NM_003264.3	TLR2	Homo sapiens toll-like receptor 2 (TLR2), mRNA.	7.44	7.66	1.16	0.0149
NM_018982.3	YIPF1	Homo sapiens Yip1 domain family, member 1 (YIPF1), mRNA.	8.89	9.10	1.16	0.0256
NM_015150.1	RAFTLIN	Homo sapiens raftlin, lipid raft linker 1 (RFTN1), mRNA.	9.33	9.54	1.16	0.0458
NM_003896.2	ST3GAL5	Homo sapiens ST3 beta-galactoside alpha-2,3-sialyltransferase 5 (ST3GAL5), mRNA.	8.64	8.85	1.16	0.0470
NM_025144.2	ALPK1	Homo sapiens alpha-kinase 1 (ALPK1), mRNA.	7.73	7.94	1.16	0.0457
NM_022833.2	C9ORF88	Homo sapiens chromosome 9 open reading frame 88 (C9orf88), mRNA.	10.72	10.93	1.16	0.0454
NM_014063.5	DBNL	Homo sapiens drebrin-like (DBNL), transcript variant 1, mRNA.	11.68	11.89	1.16	0.0375
NM_007200.3	AKAP13	Homo sapiens A kinase (PRKA) anchor protein 13 (AKAP13), transcript variant 2, mRNA.	10.54	10.74	1.16	0.0422
NM_015219.2	EXOC7	Homo sapiens exocyst complex component 7 (EXOC7), transcript variant 2, mRNA.	8.83	9.04	1.16	0.0102
NM_018326.2	GIMAP4	Homo sapiens GTPase, IMAP family member 4 (GIMAP4), mRNA.	12.37	12.58	1.16	0.0388
NM_032804.5	C10ORF22	Homo sapiens chromosome 10 open reading frame 22 (C10orf22), mRNA.	7.59	7.80	1.15	0.0183
NM_001259.5	CDK6	Homo sapiens cyclin-dependent kinase 6 (CDK6), mRNA.	6.90	7.10	1.15	0.0104
NM_001014431.1	AKT1	Homo sapiens v-akt murine thymoma viral oncogene homolog 1 (AKT1), transcript variant 3, mRNA.	9.71	9.91	1.15	0.0370
NM_007061.3	CDC42EP1	Homo sapiens CDC42 effector protein (Rho GTPase binding) 1 (CDC42EP1), transcript variant 2, mRNA.	6.88	7.08	1.15	0.0447
NM_016063.1	HDDC2	Homo sapiens HD domain containing 2 (HDDC2), mRNA.	9.06	9.26	1.15	0.0372
NM_176787.3	PIGN	Homo sapiens phosphatidylinositol glycan anchor biosynthesis, class N (PIGN), transcript variant 1, mRNA.	7.39	7.59	1.15	0.0280
NM_007165.4	SF3A2	Homo sapiens splicing factor 3a, subunit 2, 66kDa (SF3A2), mRNA.	9.10	9.30	1.15	0.0476
NM_003481.1	USP5	Homo sapiens ubiquitin specific peptidase 5 (isopeptidase T) (USP5), mRNA.	8.46	8.67	1.15	0.0317
NM_180981.1	MRPL52	Homo sapiens mitochondrial ribosomal protein L52 (MRPL52), nuclear gene encoding mitochondrial protein, transcript variant 2, mRNA.	7.71	7.91	1.15	0.0206
NM_002068.1	GNA15	Homo sapiens guanine nucleotide binding protein (G protein), alpha 15 (Gq class) (GNA15), mRNA.	9.38	9.59	1.15	0.0373
NM_005481.2	THRAP5	Homo sapiens mediator complex subunit 16 (MED16), mRNA.	8.80	9.00	1.15	0.0334
NM_080686.1	BAT2	Homo sapiens HLA-B associated transcript 2 (BAT2), transcript variant 1, mRNA.	8.10	8.30	1.15	0.0235
NM_001560.2	IL13RA1	Homo sapiens interleukin 13 receptor, alpha 1 (IL13RA1), mRNA.	10.73	10.93	1.15	0.0369
NM_000628.3	IL10RB	Homo sapiens interleukin 10 receptor, beta (IL10RB), mRNA.	11.01	11.20	1.15	0.0442
NM_024841.2	FLJ14213	Homo sapiens hypothetical protein FLJ14213 (FLJ14213), mRNA.	6.95	7.15	1.15	0.0120
NM_006761.3	YWHAE	Homo sapiens tyrosine 3-monooxygenase/tryptophan 5-monooxygenase activation protein, epsilon polypeptide (YWHAE), mRNA.	7.22	7.42	1.14	0.0416
NM_003809.2	TNFSF12	Homo sapiens tumor necrosis factor (ligand) superfamily, member 12 (TNFSF12), mRNA.	9.15	9.34	1.14	0.0493
NM_138499.2	PWWP2	Homo sapiens PWWP domain containing 2 (PWWP2), mRNA.	7.65	7.84	1.14	0.0187
NM_025078.3	PQLC1	Homo sapiens PQ loop repeat containing 1 (PQLC1), mRNA.	10.49	10.69	1.14	0.0400

NM_018103.3	LRRC8D	Homo sapiens leucine rich repeat containing 8 family, member D (LRRC8D), mRNA.	7.59	7.79	1.14	0.0451
NM_014505.4	KCNMB4	Homo sapiens potassium large conductance calcium-activated channel, subfamily M, beta member 4 (KCNMB4), mRNA.	6.38	6.57	1.14	0.0047
NM_021019.3	MYL6	Homo sapiens myosin, light chain 6, alkali, smooth muscle and non-muscle (MYL6), transcript variant 1, mRNA.	13.26	13.45	1.14	0.0294
NM_024422.2	DSC2	Homo sapiens desmocollin 2 (DSC2), transcript variant Dsc2a, mRNA.	6.56	6.75	1.14	0.0165
NM_030914.1	C9ORF74	Homo sapiens ubiquitin related modifier 1 homolog (S. cerevisiae) (URM1), mRNA.	9.46	9.65	1.14	0.0353
NM_004924.3	ACTN4	Homo sapiens actinin, alpha 4 (ACTN4), mRNA.	8.80	8.99	1.14	0.0360
NM_138578.1	BCL2L1	Homo sapiens BCL2-like 1 (BCL2L1), nuclear gene encoding mitochondrial protein, transcript variant 1, mRNA.	8.03	8.21	1.14	0.0401
NM_001101.2	ACTB	Homo sapiens actin, beta (ACTB), mRNA.	14.26	14.45	1.14	0.0374
NM_052875.2	VPS26B	Homo sapiens vacuolar protein sorting 26 homolog B (yeast) (VPS26B), mRNA.	7.75	7.94	1.14	0.0292
NM_198540.2	B3GALT7	Homo sapiens UDP-GlcNAc:betaGal beta-1,3-N-acetylglucosaminyltransferase 8 (B3GNT8), mRNA.	6.95	7.13	1.14	0.0163
NM_180991.4	SLCO4C1	Homo sapiens solute carrier organic anion transporter family, member 4C1 (SLCO4C1), mRNA.	6.35	6.53	1.13	0.0005
NM_183041.1	DTNBP1	Homo sapiens dystrobrevin binding protein 1 (DTNBP1), transcript variant 3, mRNA.	7.30	7.48	1.13	0.0310
NM_003127.1	SPTAN1	Homo sapiens spectrin, alpha, non-erythrocytic 1 (alpha-fodrin) (SPTAN1), mRNA.	8.14	8.32	1.13	0.0440
NM_018174.4	BPY2IP1	Homo sapiens microtubule-associated protein 1S (MAP1S), mRNA.	7.45	7.64	1.13	0.0084
NM_014712.1	SETD1A	Homo sapiens SET domain containing 1A (SETD1A), mRNA.	7.86	8.04	1.13	0.0144
NM_012095.4	AP3M1	Homo sapiens adaptor-related protein complex 3, mu 1 subunit (AP3M1), transcript variant 2, mRNA.	8.19	8.37	1.13	0.0257
NM_015459.3	DKFZP564J0863	Homo sapiens DKFZP564J0863 protein (DKFZP564J0863), mRNA.	7.96	8.14	1.13	0.0267
NM_022048.2	CSNK1G1	Homo sapiens casein kinase 1, gamma 1 (CSNK1G1), transcript variant 2, mRNA.	7.75	7.93	1.13	0.0330
NM_020451.2	SEPN1	Homo sapiens selenoprotein N, 1 (SEPN1), transcript variant 1, mRNA.	6.91	7.09	1.13	0.0152
NM_014800.8	ELMO1	Homo sapiens engulfment and cell motility 1 (ced-12 homolog, C. elegans) (ELMO1), transcript variant 1, mRNA.	8.18	8.36	1.13	0.0243
NM_000675.3	ADORA2A	Homo sapiens adenosine A2a receptor (ADORA2A), mRNA.	6.77	6.94	1.13	0.0249
NM_018054.4	ARHGAP17	Homo sapiens Rho GTPase activating protein 17 (ARHGAP17), transcript variant 2, mRNA.	7.46	7.63	1.13	0.0333
NM_015609.1	C1ORF144	Homo sapiens chromosome 1 open reading frame 144 (C1orf144), mRNA.	8.55	8.72	1.12	0.0443
NM_006116.2	MAP3K7IP1	Homo sapiens mitogen-activated protein kinase kinase kinase 7 interacting protein 1 (MAP3K7IP1), transcript variant alpha, mRNA.	7.60	7.77	1.12	0.0282
NM_024293.2	C2ORF17	Homo sapiens chromosome 2 open reading frame 17 (C2orf17), mRNA.	7.85	8.02	1.12	0.0456
NM_001037633.1	SIL1	Homo sapiens SIL1 homolog, endoplasmic reticulum chaperone (S. cerevisiae) (SIL1), transcript variant 1, mRNA.	7.55	7.72	1.12	0.0488
NM_007259.2	VPS45A	Homo sapiens vacuolar protein sorting 45A (yeast) (VPS45A), mRNA.	8.58	8.75	1.12	0.0482
NM_012320.3	LYPLA3	Homo sapiens lysophospholipase 3 (lysosomal phospholipase A2) (LYPLA3), mRNA.	7.05	7.22	1.12	0.0218
NM_033446.1	C9ORF28	Homo sapiens family with sequence similarity 125, member B (FAM125B), transcript variant 1, mRNA.	7.13	7.30	1.12	0.0344
NM_032730.3	RTN4IP1	Homo sapiens reticulon 4 interacting protein 1 (RTN4IP1), nuclear gene encoding mitochondrial protein, mRNA.	6.85	7.01	1.12	0.0124
XM_946079.1	C7ORF20	PREDICTED: Homo sapiens chromosome 7 open reading frame 20, transcript variant 6 (C7orf20), mRNA.	7.25	7.41	1.12	0.0490
NM_024517.1	PHF2	Homo sapiens PHD finger protein 2 (PHF2), transcript variant 2, mRNA.	8.08	8.24	1.12	0.0418
NM_030805.1	LMAN2L	Homo sapiens lectin, mannose-binding 2-like (LMAN2L), mRNA.	7.34	7.50	1.12	0.0473
NM_024025.1	DUSP26	Homo sapiens dual specificity phosphatase 26 (putative) (DUSP26), mRNA.	7.25	7.41	1.12	0.0406
NM_183425.1	RBM38	Homo sapiens RNA binding motif protein 38 (RBM38), transcript variant 2, mRNA.	6.83	6.99	1.12	0.0324
NM_145800.2	Sep-06	Homo sapiens septin 6 (SEPT6), transcript variant III, mRNA.	6.94	7.10	1.11	0.0360
NM_005477.1	HCN4	Homo sapiens hyperpolarization activated cyclic nucleotide-gated potassium channel 4 (HCN4), mRNA.	6.44	6.59	1.11	0.0353
NM_139021.1	MAPK15	Homo sapiens mitogen-activated protein kinase 15 (MAPK15), mRNA.	6.52	6.67	1.11	0.0318
NM_202468.1	GIPC1	Homo sapiens GIPC PDZ domain containing family, member 1 (GIPC1), transcript variant 3, mRNA.	6.68	6.83	1.11	0.0167
NM_004960.2	FUS	Homo sapiens fusion (involved in t(12;16) in malignant liposarcoma) (FUS), mRNA.	6.79	6.94	1.11	0.0119
NM_020314.3	MGC16824	Homo sapiens esophageal cancer associated protein (MGC16824), mRNA.	6.71	6.86	1.11	0.0248
NM_004798.2	KIF3B	Homo sapiens kinesin family member 3B (KIF3B), mRNA.	7.74	7.89	1.11	0.0458
NM_052848.1	MGC20255	Homo sapiens coiled-coil domain containing 97 (CCDC97), mRNA.	7.51	7.66	1.11	0.0421
NM_018243.2	Sep-11	Homo sapiens septin 11 (SEPT11), mRNA.	7.02	7.16	1.11	0.0307
NM_001001939.1	PCBD1	Homo sapiens 6-pyruvoyl-tetrahydropterin synthase/dimerization cofactor of hepatocyte nuclear factor 1 alpha (TCF1) (PCBD1), transcript variant 2, r	7.40	7.54	1.11	0.0474
NM_003816.2	ADAM9	Homo sapiens ADAM metallopeptidase domain 9 (meltrin gamma) (ADAM9), transcript variant 1, mRNA.	6.89	7.04	1.10	0.0312
NM_005535.1	IL12RB1	Homo sapiens interleukin 12 receptor, beta 1 (IL12RB1), transcript variant 1, mRNA.	6.57	6.71	1.10	0.0057
NM_019843.2	EIF4ENIF1	Homo sapiens eukaryotic translation initiation factor 4E nuclear import factor 1 (EIF4ENIF1), mRNA.	7.29	7.43	1.10	0.0377
NM_004957.4	FPGS	Homo sapiens folylpolyglutamate synthase (FPGS), nuclear gene encoding mitochondrial protein, transcript variant 1, mRNA.	6.45	6.59	1.10	0.0056
NM_013347.1	RPA4	Homo sapiens replication protein A4, 34kDa (RPA4), mRNA.	6.73	6.87	1.10	0.0394
NM_014508.2	APOBEC3C	Homo sapiens apolipoprotein B mRNA editing enzyme, catalytic polypeptide-like 3C (APOBEC3C), mRNA.	6.99	7.13	1.10	0.0333
NM_031466.3	NIBP	Homo sapiens NIK and IKK{beta} binding protein (NIBP), mRNA.	7.10	7.24	1.10	0.0318
NM_213568.1	SLC39A3	Homo sapiens solute carrier family 39 (zinc transporter), member 3 (SLC39A3), transcript variant 2, mRNA.	6.43	6.56	1.10	0.0054
NM_001001795.1	MGC70857	Homo sapiens similar to RIKEN cDNA C030006K11 gene (MGC70857), mRNA.	7.10	7.24	1.10	0.0382
NM_018645.3	HES6	Homo sapiens hairy and enhancer of split 6 (Drosophila) (HES6), mRNA.	6.77	6.90	1.10	0.0325
NM_147202.1	C9ORF25	Homo sapiens chromosome 9 open reading frame 25 (C9orf25), mRNA.	6.79	6.92	1.10	0.0166
NM_152267.2	RNF185	Homo sapiens ring finger protein 185 (RNF185), mRNA.	7.07	7.20	1.10	0.0429
NM_000297.2	PKD2	Homo sapiens polycystic kidney disease 2 (autosomal dominant) (PKD2), mRNA.	6.94	7.07	1.10	0.0425
NM_032932.3	RAB11FIP4	Homo sapiens RAB11 family interacting protein 4 (class II) (RAB11FIP4), mRNA.	6.54	6.67	1.09	0.0439
NM_006068.2	TLR6	Homo sapiens toll-like receptor 6 (TLR6), mRNA.	6.69	6.82	1.09	0.0214
NM_005311.3	GRB10	Homo sapiens growth factor receptor-bound protein 10 (GRB10), transcript variant 1, mRNA.	6.76	6.89	1.09	0.0328
NM_000755.2	CRAT	Homo sapiens carnitine acetyltransferase (CRAT), transcript variant 1, mRNA.	6.46	6.59	1.09	0.0126
NM_032222.1	FLJ22374	Homo sapiens hypothetical protein FLJ22374 (FLJ22374), mRNA.	6.44	6.56	1.09	0.0115
NM_033211.2	LOC90355	Homo sapiens chromosome 5 open reading frame 30 (C5orf30), mRNA.	6.47	6.60	1.09	0.0160
NM_015441.1	OLFML2B	Homo sapiens olfactomedin-like 2B (OLFML2B), mRNA.	6.59	6.71	1.09	0.0394
NM_001007232.1	INCA	Homo sapiens inhibitory caspase recruitment domain (CARD) protein (INCA), mRNA.	6.55	6.67	1.09	0.0214
NM_022086.6	ELMO2	Homo sapiens engulfment and cell motility 2 (ced-12 homolog, C. elegans) (ELMO2), transcript variant 2, mRNA.	6.47	6.59	1.09	0.0298
NM_014925.2	R3HDM2	Homo sapiens R3H domain containing 2 (R3HDM2), mRNA.	6.85	6.97	1.08	0.0433
NM_138422.1	LOC113179	Homo sapiens tRNA-specific adenosine deaminase 3 (ADAT3), mRNA.	6.54	6.65	1.08	0.0121

NM_001042535.1	CENTG3	Homo sapiens centaurin, gamma 3 (CENTG3), transcript variant 2, mRNA.	6.48	6.60	1.08	0.0202
NM_001012288.1	CRLF2	Homo sapiens cytokine receptor-like factor 2 (CRLF2), transcript variant 2, mRNA.	6.48	6.60	1.08	0.0353
NM_001031854.1	LOC390110	Homo sapiens hypothetical protein (LOC390110), mRNA.	6.43	6.54	1.08	0.0184
NM_001149.2	ANK3	Homo sapiens ankyrin 3, node of Ranvier (ankyrin G) (ANK3), transcript variant 2, mRNA.	6.39	6.51	1.08	0.0096
NM_152455.2	ZNF690	Homo sapiens zinc finger protein 690 (ZNF690), mRNA.	6.38	6.49	1.08	0.0257
NM_004565.1	PEX14	Homo sapiens peroxisomal biogenesis factor 14 (PEX14), mRNA.	6.46	6.56	1.07	0.0400
NM_022896.1	LPIN3	Homo sapiens lipin 3 (LPIN3), mRNA.	6.45	6.56	1.07	0.0415
NM_145344.1	APOL1	Homo sapiens apolipoprotein L, 1 (APOL1), transcript variant 3, mRNA.	6.50	6.60	1.07	0.0378
NM_001040694.1	INCENP	Homo sapiens inner centromere protein antigens 135/155kDa (INCENP), transcript variant 1, mRNA.	6.52	6.61	1.07	0.0460
NM_020341.2	PAK7	Homo sapiens p21(CDKN1A)-activated kinase 7 (PAK7), transcript variant 1, mRNA.	6.44	6.53	1.06	0.0423
NM_020536.2	CSRP2BP	Homo sapiens CSRP2 binding protein (CSRP2BP), transcript variant 1, mRNA.	6.43	6.52	1.06	0.0391
NM_017793.1	RPP25	Homo sapiens ribonuclease P 25kDa subunit (RPP25), mRNA.	6.50	6.59	1.06	0.0461
NM_176795.2	HRAS	Homo sapiens v-Ha-ras Harvey rat sarcoma viral oncogene homolog (HRAS), transcript variant 2, mRNA.	6.40	6.49	1.06	0.0465
**DOWNREGULATED**						
NM_000518.4	HBB	Homo sapiens hemoglobin, beta (HBB), mRNA.	11.48	9.73	0.30	0.0010
NM_000517.3	HBA2	Homo sapiens hemoglobin, alpha 2 (HBA2), mRNA.	9.85	8.43	0.38	0.0026
XM_936120.1	HLA-DQA1	PREDICTED: Homo sapiens major histocompatibility complex, class II, DQ alpha 1, transcript variant 2 (HLA-DQA1), mRNA.	11.71	10.64	0.47	0.0056
NM_000584.2	IL8	Homo sapiens interleukin 8 (IL8), mRNA.	7.79	7.01	0.58	0.0007
NM_005252.2	FOS	Homo sapiens v-fos FBJ murine osteosarcoma viral oncogene homolog (FOS), mRNA.	9.72	9.00	0.61	0.0116
NM_006732.1	FOSB	Homo sapiens FBJ murine osteosarcoma viral oncogene homolog B (FOSB), mRNA.	8.41	7.69	0.61	0.0000
NM_001964.2	EGR1	Homo sapiens early growth response 1 (EGR1), mRNA.	8.12	7.49	0.65	0.0076
NM_004417.2	DUSP1	Homo sapiens dual specificity phosphatase 1 (DUSP1), mRNA.	11.75	11.19	0.68	0.0030
NM_004666.1	VNN1	Homo sapiens vanin 1 (VNN1), mRNA.	8.75	8.19	0.68	0.0028
NM_005502.2	ABCA1	Homo sapiens ATP-binding cassette, sub-family A (ABC1), member 1 (ABCA1), mRNA.	8.76	8.26	0.71	0.0085
NM_020152.2	C21ORF7	Homo sapiens chromosome 21 open reading frame 7 (C21orf7), mRNA.	8.44	7.94	0.71	0.0092
NM_017933.3	FLJ20701	Homo sapiens phosphotyrosine interaction domain containing 1 (PID1), mRNA.	8.91	8.45	0.72	0.0008
NM_002612.2	PDK4	Homo sapiens pyruvate dehydrogenase kinase, isozyme 4 (PDK4), mRNA.	9.31	8.87	0.74	0.0166
NM_021732.1	AVPI1	Homo sapiens arginine vasopressin-induced 1 (AVPI1), mRNA.	8.11	7.67	0.74	0.0055
NM_014887.1	PFAAP5	Homo sapiens phosphonoformate immuno-associated protein 5 (PFAAP5), mRNA.	8.70	8.27	0.74	0.0004
NM_002201.4	ISG20	Homo sapiens interferon stimulated exonuclease gene 20kDa (ISG20), mRNA.	9.39	8.97	0.75	0.0057
NM_015263.1	DMXL2	Homo sapiens Dmx-like 2 (DMXL2), mRNA.	9.79	9.38	0.76	0.0134
NM_003937.2	KYNU	Homo sapiens kynureninase (L-kynurenine hydrolase) (KYNU), transcript variant 1, mRNA.	9.76	9.35	0.76	0.0028
NM_138373.3	MYADM	Homo sapiens myeloid-associated differentiation marker (MYADM), transcript variant 2, mRNA.	8.76	8.36	0.76	0.0025
NM_006343.2	MERTK	Homo sapiens c-mer proto-oncogene tyrosine kinase (MERTK), mRNA.	8.55	8.15	0.76	0.0230
NM_001001437.3	CCL3L3	Homo sapiens chemokine (C-C motif) ligand 3-like 3 (CCL3L3), mRNA.	7.05	6.67	0.77	0.0193
NM_014468.2	VENTX	Homo sapiens VENT homeobox homolog (Xenopus laevis) (VENTX), mRNA.	9.38	9.00	0.77	0.0052
NM_002982.3	CCL2	Homo sapiens chemokine (C-C motif) ligand 2 (CCL2), mRNA.	7.26	6.88	0.77	0.0081
NM_001004305.1	LOC284757	Homo sapiens hypothetical protein LOC284757 (LOC284757), mRNA.	6.91	6.54	0.77	0.0038
NM_017652.1	ZNF586	Homo sapiens zinc finger protein 586 (ZNF586), mRNA.	8.81	8.46	0.78	0.0043
NM_003663.3	CGGBP1	Homo sapiens CGG triplet repeat binding protein 1 (CGGBP1), transcript variant 2, mRNA.	9.60	9.25	0.78	0.0052
NM_003937.2	KYNU	Homo sapiens kynureninase (L-kynurenine hydrolase) (KYNU), transcript variant 1, mRNA.	10.22	9.87	0.79	0.0015
NM_054114.3	TAGAP	Homo sapiens T-cell activation GTPase activating protein (TAGAP), transcript variant 2, mRNA.	8.37	8.03	0.79	0.0175
NM_001641.2	APEX1	Homo sapiens APEX nuclease (multifunctional DNA repair enzyme) 1 (APEX1), transcript variant 1, mRNA.	9.25	8.91	0.79	0.0016
NM_001020820.1	MYADM	Homo sapiens myeloid-associated differentiation marker (MYADM), transcript variant 4, mRNA.	12.04	11.71	0.79	0.0195
NM_014585.3	SLC40A1	Homo sapiens solute carrier family 40 (iron-regulated transporter), member 1 (SLC40A1), mRNA.	9.65	9.32	0.79	0.0203
NM_021960.3	MCL1	Homo sapiens myeloid cell leukemia sequence 1 (BCL2-related) (MCL1), transcript variant 1, mRNA.	10.25	9.91	0.79	0.0259
NM_002120.2	HLA-DOB	Homo sapiens major histocompatibility complex, class II, DO beta (HLA-DOB), mRNA.	7.17	6.84	0.79	0.0173
NM_022495.3	C14ORF135	Homo sapiens chromosome 14 open reading frame 135 (C14orf135), mRNA.	8.71	8.38	0.80	0.0037
NM_020070.2	IGLL1	Homo sapiens immunoglobulin lambda-like polypeptide 1 (IGLL1), transcript variant 1, mRNA.	7.15	6.82	0.80	0.0126
NM_002600.3	PDE4B	Homo sapiens phosphodiesterase 4B, cAMP-specific (phosphodiesterase E4 dunce homolog, Drosophila) (PDE4B), transcript variant a, mRNA.	7.98	7.65	0.80	0.0083
NM_001023582.1	RPGR	Homo sapiens retinitis pigmentosa GTPase regulator (RPGR), transcript variant B, mRNA.	7.37	7.04	0.80	0.0015
NM_001530.2	HIF1A	Homo sapiens hypoxia-inducible factor 1, alpha subunit (basic helix-loop-helix transcription factor) (HIF1A), transcript variant 1, mRNA.	7.86	7.53	0.80	0.0102
NM_001080498.1	EMR4	Homo sapiens egf-like module containing, mucin-like, hormone receptor-like 4 (EMR4), mRNA.	6.78	6.46	0.80	0.0227
NM_032472.3	PPIL3	Homo sapiens peptidylprolyl isomerase (cyclophilin)-like 3 (PPIL3), transcript variant PPIL3a, mRNA.	10.19	9.87	0.80	0.0438
NM_018398.2	CACNA2D3	Homo sapiens calcium channel, voltage-dependent, alpha 2/delta 3 subunit (CACNA2D3), mRNA.	8.44	8.12	0.80	0.0140
NM_153322.1	PMP22	Homo sapiens peripheral myelin protein 22 (PMP22), transcript variant 3, mRNA.	7.00	6.69	0.80	0.0086
NM_152716.1	FLJ36874	Homo sapiens FLJ36874 protein (FLJ36874), mRNA.	10.49	10.18	0.81	0.0100
NM_001001974.1	PLEKHA1	Homo sapiens pleckstrin homology domain containing, family A (phosphoinositide binding specific) member 1 (PLEKHA1), transcript variant 2, mRNA	7.92	7.60	0.81	0.0034
NM_001080392.1	KIAA1147	Homo sapiens KIAA1147 (KIAA1147), mRNA.	9.03	8.73	0.81	0.0429
NM_003913.3	PRPF4B	Homo sapiens PRP4 pre-mRNA processing factor 4 homolog B (yeast) (PRPF4B), transcript variant 1, mRNA.	8.67	8.36	0.81	0.0137
NM_014059.1	RGC32	Homo sapiens response gene to complement 32 (RGC32), mRNA.	7.71	7.41	0.81	0.0036
NM_032308.1	RPAIN	Homo sapiens RPA interacting protein (RPAIN), transcript variant 2, mRNA.	10.37	10.07	0.81	0.0175
NM_014886.2	TINP1	Homo sapiens TGF beta-inducible nuclear protein 1 (TINP1), mRNA.	11.65	11.35	0.81	0.0306
NM_005087.2	FXR1	Homo sapiens fragile X mental retardation, autosomal homolog 1 (FXR1), transcript variant 1, mRNA.	8.97	8.67	0.81	0.0034
NM_002157.1	HSPE1	Homo sapiens heat shock 10kDa protein 1 (chaperonin 10) (HSPE1), mRNA.	8.36	8.07	0.82	0.0042
NM_005463.2	HNRPDL	Homo sapiens heterogeneous nuclear ribonucleoprotein D-like (HNRPDL), transcript variant 1, mRNA.	9.72	9.43	0.82	0.0128
NM_004986.2	KTN1	Homo sapiens kinectin 1 (kinesin receptor) (KTN1), transcript variant 4, mRNA.	8.83	8.55	0.82	0.0043
NM_001079539.1	XBP1	Homo sapiens X-box binding protein 1 (XBP1), transcript variant 2, mRNA.	10.64	10.36	0.82	0.0369
NM_018664.1	SNFT	Homo sapiens Jun dimerization protein p21SNFT (SNFT), mRNA.	8.15	7.86	0.82	0.0215

NM_002101.3	GYPC	Homo sapiens glycophorin C (Gerbich blood group) (GYPC), transcript variant 1, mRNA.	8.45	8.16	0.82	0.0063
NR_002166.1	SEDLP	Homo sapiens spondyloepiphyseal dysplasia, late, pseudogene (SEDLP) on chromosome 19.	6.98	6.70	0.82	0.0003
NM_018643.2	TREM1	Homo sapiens triggering receptor expressed on myeloid cells 1 (TREM1), mRNA.	7.76	7.48	0.82	0.0206
NM_175738.3	RAB37	Homo sapiens RAB37, member RAS oncogene family (RAB37), transcript variant 3, mRNA.	8.89	8.62	0.82	0.0178
NM_016316.1	REV1L	Homo sapiens REV1-like (yeast) (REV1L), mRNA.	8.66	8.38	0.82	0.0022
NM_004506.2	HSF2	Homo sapiens heat shock transcription factor 2 (HSF2), mRNA.	7.09	6.82	0.83	0.0008
NM_173343.1	IL1R2	Homo sapiens interleukin 1 receptor, type II (IL1R2), transcript variant 2, mRNA.	7.61	7.33	0.83	0.0442
NM_001012734.1	AGPAT4	Homo sapiens 1-acylglycerol-3-phosphate O-acyltransferase 4 (lysophosphatidic acid acyltransferase, delta) (AGPAT4), transcript variant 3, mRNA.	8.22	7.94	0.83	0.0189
NM_023039.2	ANKRA2	Homo sapiens ankyrin repeat, family A (RFXANK-like), 2 (ANKRA2), mRNA.	8.82	8.55	0.83	0.0186
NM_207304.1	MBNL2	Homo sapiens muscleblind-like 2 (Drosophila) (MBNL2), transcript variant 3, mRNA.	8.99	8.72	0.83	0.0168
NM_015308.1	FNBP4	Homo sapiens formin binding protein 4 (FNBP4), mRNA.	11.03	10.77	0.83	0.0377
NM_001024070.1	GCH1	Homo sapiens GTP cyclohydrolase 1 (dopa-responsive dystonia) (GCH1), transcript variant 3, mRNA.	7.78	7.52	0.83	0.0436
NM_139075.1	TPCN2	Homo sapiens two pore segment channel 2 (TPCN2), mRNA.	8.83	8.56	0.83	0.0176
NR_002944.2	HNRPA1L-2	Homo sapiens heterogeneous nuclear ribonucleoprotein A1 pseudogene (HNRPA1L-2) on chromosome 19.	8.68	8.42	0.83	0.0254
NM_001010.2	RPS6	Homo sapiens ribosomal protein S6 (RPS6), mRNA.	12.41	12.15	0.83	0.0478
NM_080649.1	APEX1	Homo sapiens APEX nuclease (multifunctional DNA repair enzyme) 1 (APEX1), transcript variant 3, mRNA.	10.71	10.45	0.84	0.0428
NM_203433.1	DSCR2	Homo sapiens Down syndrome critical region gene 2 (DSCR2), transcript variant 2, mRNA.	7.75	7.49	0.84	0.0099
NM_001621.3	AHR	Homo sapiens aryl hydrocarbon receptor (AHR), mRNA.	7.53	7.28	0.84	0.0350
NM_144778.2	MBNL2	Homo sapiens muscleblind-like 2 (Drosophila) (MBNL2), transcript variant 1, mRNA.	8.09	7.84	0.84	0.0079
NM_005792.1	MPHOSPH6	Homo sapiens M-phase phosphoprotein 6 (MPHOSPH6), mRNA.	6.75	6.50	0.84	0.0000
NM_001280.1	CIRBP	Homo sapiens cold inducible RNA binding protein (CIRBP), mRNA.	9.76	9.50	0.84	0.0241
NM_138810.2	TAGAP	Homo sapiens T-cell activation GTPase activating protein (TAGAP), transcript variant 3, mRNA.	8.10	7.85	0.84	0.0421
NM_032592.1	PHACS	Homo sapiens 1-aminocyclopropane-1-carboxylate synthase (PHACS), mRNA.	7.53	7.28	0.84	0.0368
NM_005385.3	NKTR	Homo sapiens natural killer-tumor recognition sequence (NKTR), mRNA.	9.96	9.71	0.84	0.0206
NM_001078645.1	CDC16	Homo sapiens cell division cycle 16 homolog (S. cerevisiae) (CDC16), transcript variant 2, mRNA.	10.58	10.33	0.84	0.0406
NM_139343.1	BIN1	Homo sapiens bridging integrator 1 (BIN1), transcript variant 1, mRNA.	7.14	6.90	0.84	0.0179
NM_016818.2	ABCG1	Homo sapiens ATP-binding cassette, sub-family G (WHITE), member 1 (ABCG1), transcript variant 2, mRNA.	6.71	6.46	0.84	0.0107
NM_016006.3	ABHD5	Homo sapiens abhydrolase domain containing 5 (ABHD5), mRNA.	8.15	7.90	0.84	0.0202
NM_016474.3	C3ORF19	Homo sapiens chromosome 3 open reading frame 19 (C3orf19), mRNA.	9.77	9.52	0.84	0.0459
NM_015225.1	KIAA0367	Homo sapiens KIAA0367 (KIAA0367), mRNA.	6.83	6.58	0.84	0.0273
NM_016594.1	FKBP11	Homo sapiens FK506 binding protein 11, 19 kDa (FKBP11), mRNA.	8.27	8.03	0.84	0.0107
NM_021078.1	GCN5L2	Homo sapiens GCN5 general control of amino-acid synthesis 5-like 2 (yeast) (GCN5L2), mRNA.	8.96	8.71	0.84	0.0161
NM_018032.3	LUC7L	Homo sapiens LUC7-like (S. cerevisiae) (LUC7L), transcript variant 1, mRNA.	8.25	8.01	0.84	0.0115
NM_003972.2	BTAF1	Homo sapiens BTAF1 RNA polymerase II, B-TFIID transcription factor-associated, 170kDa (Mot1 homolog, S. cerevisiae) (BTAF1), mRNA.	10.08	9.84	0.84	0.0253
NM_004848.1	C1ORF38	Homo sapiens chromosome 1 open reading frame 38 (C1orf38), mRNA.	8.05	7.81	0.85	0.0070
NM_006321.1	ARIH2	Homo sapiens ariadne homolog 2 (Drosophila) (ARIH2), mRNA.	9.54	9.30	0.85	0.0313
NM_015130.1	TBC1D9	Homo sapiens TBC1 domain family, member 9 (with GRAM domain) (TBC1D9), mRNA.	9.44	9.20	0.85	0.0263
NM_012433.2	SF3B1	Homo sapiens splicing factor 3b, subunit 1, 155kDa (SF3B1), transcript variant 1, mRNA.	10.70	10.46	0.85	0.0200
NM_007079.2	PTP4A3	Homo sapiens protein tyrosine phosphatase type IVA, member 3 (PTP4A3), transcript variant 2, mRNA.	6.93	6.69	0.85	0.0080
NM_006779.2	CDC42EP2	Homo sapiens CDC42 effector protein (Rho GTPase binding) 2 (CDC42EP2), mRNA.	8.22	7.99	0.85	0.0235
NM_001006623.1	WDR33	Homo sapiens WD repeat domain 33 (WDR33), transcript variant 3, mRNA.	7.86	7.62	0.85	0.0288
NM_001033853.1	RPL3	Homo sapiens ribosomal protein L3 (RPL3), transcript variant 2, mRNA.	12.07	11.83	0.85	0.0411
NM_004137.2	KCNMB1	Homo sapiens potassium large conductance calcium-activated channel, subfamily M, beta member 1 (KCNMB1), mRNA.	9.67	9.44	0.85	0.0359
NM_006469.4	IVNS1ABP	Homo sapiens influenza virus NS1A binding protein (IVNS1ABP), mRNA.	8.72	8.48	0.85	0.0315
NM_024558.1	C14ORF138	Homo sapiens chromosome 14 open reading frame 138 (C14orf138), mRNA.	7.64	7.40	0.85	0.0368
NM_153682.1	DSCR5	Homo sapiens Down syndrome critical region gene 5 (DSCR5), transcript variant 2, mRNA.	7.53	7.30	0.85	0.0267
NM_004641.2	MLLT10	Homo sapiens myeloid/lymphoid or mixed-lineage leukemia (trithorax homolog, Drosophila); translocated to, 10 (MLLT10), transcript variant 1, mRNA	8.82	8.59	0.85	0.0117
NM_024692.3	RSNL2	Homo sapiens CAP-GLY domain containing linker protein family, member 4 (CLIP4), mRNA.	8.32	8.09	0.85	0.0209
NM_016463.5	CXXC5	Homo sapiens CXXC finger 5 (CXXC5), mRNA.	8.48	8.25	0.86	0.0340
NM_181873.1	MTMR11	Homo sapiens myotubularin related protein 11 (MTMR11), transcript variant 2, mRNA.	10.88	10.65	0.86	0.0399
NM_172364.3	CACNA2D4	Homo sapiens calcium channel, voltage-dependent, alpha 2/delta subunit 4 (CACNA2D4), transcript variant 1, mRNA.	8.02	7.80	0.86	0.0318
NM_000256.2	MYBPC3	Homo sapiens myosin binding protein C, cardiac (MYBPC3), mRNA.	8.23	8.01	0.86	0.0347
NM_002285.2	AFF3	Homo sapiens AF4/FMR2 family, member 3 (AFF3), transcript variant 1, mRNA.	6.88	6.66	0.86	0.0035
NM_001441.1	FAAH	Homo sapiens fatty acid amide hydrolase (FAAH), mRNA.	7.26	7.04	0.86	0.0234
NM_006874.2	ELF2	Homo sapiens E74-like factor 2 (ets domain transcription factor) (ELF2), transcript variant 2, mRNA.	9.10	8.88	0.86	0.0427
NM_133173.2	APBB3	Homo sapiens amyloid beta (A4) precursor protein-binding, family B, member 3 (APBB3), transcript variant 2, mRNA.	8.66	8.44	0.86	0.0091
NM_005646.2	TARBP1	Homo sapiens Tar (HIV-1) RNA binding protein 1 (TARBP1), mRNA.	7.20	6.98	0.86	0.0122
NM_020472.1	PIGA	Homo sapiens phosphatidylinositol glycan, class A (paroxysmal nocturnal hemoglobinuria) (PIGA), transcript variant 2, mRNA.	8.55	8.33	0.86	0.0446
NM_017426.2	NUP54	Homo sapiens nucleoporin 54kDa (NUP54), mRNA.	8.60	8.38	0.86	0.0416
NM_139353.1	TAF1C	Homo sapiens TATA box binding protein (TBP)-associated factor, RNA polymerase I, C, 110kDa (TAF1C), transcript variant 2, mRNA.	10.08	9.87	0.86	0.0359
NM_001770.3	CD19	Homo sapiens CD19 antigen (CD19), mRNA.	6.83	6.61	0.86	0.0477
NM_006469.4	IVNS1ABP	Homo sapiens influenza virus NS1A binding protein (IVNS1ABP), mRNA.	8.95	8.74	0.86	0.0432
NM_015508.2	TIPARP	Homo sapiens TCDD-inducible poly(ADP-ribose) polymerase (TIPARP), mRNA.	9.21	9.00	0.86	0.0478
NM_015151.2	DIP2A	Homo sapiens DIP2 disco-interacting protein 2 homolog A (Drosophila) (DIP2A), transcript variant 1, mRNA.	7.53	7.32	0.86	0.0129
NM_016640.3	MRPS30	Homo sapiens mitochondrial ribosomal protein S30 (MRPS30), nuclear gene encoding mitochondrial protein, mRNA.	8.94	8.73	0.86	0.0486
NM_001004307.1	MGC33556	Homo sapiens hypothetical LOC339541 (MGC33556), mRNA.	7.91	7.70	0.86	0.0326
NM_016424.3	CROP	Homo sapiens cisplatin resistance-associated overexpressed protein (CROP), transcript variant 1, mRNA.	8.28	8.07	0.86	0.0333
NM_002161.2	IARS	Homo sapiens isoleucine-tRNA synthetase (IARS), transcript variant short, mRNA.	7.40	7.19	0.86	0.0263
NM_002896.1	RBM4	Homo sapiens RNA binding motif protein 4 (RBM4), mRNA.	9.38	9.17	0.86	0.0401

NM_002598.2	PDCD2	Homo sapiens programmed cell death 2 (PDCD2), transcript variant 1, mRNA.	9.26	9.05	0.86	0.0395
NM_001017.2	RPS13	Homo sapiens ribosomal protein S13 (RPS13), mRNA.	13.05	12.84	0.86	0.0439
NM_024610.3	HSPBAP1	Homo sapiens HSPB (heat shock 27kDa) associated protein 1 (HSPBAP1), mRNA.	9.36	9.15	0.86	0.0169
NM_002983.1	CCL3	Homo sapiens chemokine (C-C motif) ligand 3 (CCL3), mRNA.	6.84	6.63	0.86	0.0270
NM_020666.2	CLK4	Homo sapiens CDC-like kinase 4 (CLK4), mRNA.	8.19	7.98	0.87	0.0217
NM_024820.2	DENND1A	Homo sapiens DENN/MADD domain containing 1A (DENND1A), transcript variant 2, mRNA.	8.35	8.14	0.87	0.0473
NM_004520.1	KIF2	Homo sapiens kinesin heavy chain member 2A (KIF2A), mRNA.	7.62	7.41	0.87	0.0430
NM_001675.2	ATF4	Homo sapiens activating transcription factor 4 (tax-responsive enhancer element B67) (ATF4), transcript variant 1, mRNA.	6.71	6.51	0.87	0.0018
NM_021178.2	CCNB1IP1	Homo sapiens cyclin B1 interacting protein 1 (CCNB1IP1), transcript variant 1, mRNA.	7.26	7.05	0.87	0.0200
NM_001023571.1	IQCB1	Homo sapiens IQ motif containing B1 (IQCB1), transcript variant 3, mRNA.	8.18	7.98	0.87	0.0467
NM_003119.2	SPG7	Homo sapiens spastic paraplegia 7 (pure and complicated autosomal recessive) (SPG7), nuclear gene encoding mitochondrial protein, transcript vari	10.46	10.26	0.87	0.0290
NM_006122.2	MAN2A2	Homo sapiens mannosidase, alpha, class 2A, member 2 (MAN2A2), mRNA.	7.77	7.57	0.87	0.0417
NM_020338.2	RAI17	Homo sapiens zinc finger, MIZ-type containing 1 (ZMIZ1), mRNA.	10.95	10.75	0.87	0.0496
NM_022828.2	YTHDC2	Homo sapiens YTH domain containing 2 (YTHDC2), mRNA.	7.22	7.02	0.87	0.0049
NM_014412.2	CACYBP	Homo sapiens calcyclin binding protein (CACYBP), transcript variant 1, mRNA.	7.36	7.16	0.87	0.0166
NM_006051.2	APBB3	Homo sapiens amyloid beta (A4) precursor protein-binding, family B, member 3 (APBB3), transcript variant 4, mRNA.	9.53	9.33	0.87	0.0354
NM_139022.2	TSPAN32	Homo sapiens tetraspanin 32 (TSPAN32), transcript variant 1, mRNA.	8.45	8.25	0.87	0.0446
NM_031284.3	ADPGK	Homo sapiens ADP-dependent glucokinase (ADPGK), mRNA.	9.81	9.61	0.87	0.0262
NM_006447.2	USP16	Homo sapiens ubiquitin specific peptidase 16 (USP16), transcript variant 1, mRNA.	8.98	8.78	0.87	0.0404
NM_052869.1	TTYH2	Homo sapiens tweety homolog 2 (Drosophila) (TTYH2), transcript variant 2, mRNA.	8.35	8.15	0.87	0.0372
NM_019600.1	KIAA1370	Homo sapiens KIAA1370 (KIAA1370), mRNA.	7.61	7.41	0.87	0.0152
NM_198055.1	ZNF42	Homo sapiens myeloid zinc finger 1 (MZF1), transcript variant 2, mRNA.	9.46	9.26	0.87	0.0297
NM_003756.1	EIF3S3	Homo sapiens eukaryotic translation initiation factor 3, subunit 3 gamma, 40kDa (EIF3S3), mRNA.	10.81	10.61	0.87	0.0480
NM_024561.3	NARG1L	Homo sapiens NMDA receptor regulated 1-like (NARG1L), transcript variant 1, mRNA.	7.34	7.15	0.87	0.0099
NM_001010909.1	C6ORF205	Homo sapiens chromosome 6 open reading frame 205 (C6orf205), mRNA.	6.89	6.69	0.87	0.0031
NM_001080498.1	EMR4	Homo sapiens egf-like module containing, mucin-like, hormone receptor-like 4 (EMR4), mRNA.	6.48	6.29	0.87	0.0172
NM_002015.2	FOXO1A	Homo sapiens forkhead box O1A (rhabdomyosarcoma) (FOXO1A), mRNA.	8.43	8.23	0.87	0.0410
NM_021209.3	CARD12	Homo sapiens NLR family, CARD domain containing 4 (NLRC4), mRNA.	8.89	8.69	0.87	0.0283
NM_003633.1	ENC1	Homo sapiens ectodermal-neural cortex (with BTB-like domain) (ENC1), mRNA.	8.51	8.32	0.87	0.0357
NR_002200.1	FTHL2	Homo sapiens ferritin, heavy polypeptide-like 2 (FTHL2) on chromosome 1.	7.57	7.37	0.87	0.0463
NM_001032287.1	NR2C1	Homo sapiens nuclear receptor subfamily 2, group C, member 1 (NR2C1), transcript variant 2, mRNA.	7.40	7.21	0.87	0.0186
NM_006190.3	ORC2L	Homo sapiens origin recognition complex, subunit 2-like (yeast) (ORC2L), mRNA.	7.66	7.47	0.88	0.0433
NM_054016.1	FUSIP1	Homo sapiens FUS interacting protein (serine/arginine-rich) 1 (FUSIP1), transcript variant 2, mRNA.	7.31	7.11	0.88	0.0127
NM_002892.2	ARID4A	Homo sapiens AT rich interactive domain 4A (RBP1-like) (ARID4A), transcript variant 1, mRNA.	8.02	7.83	0.88	0.0158
NM_030948.1	PHACTR1	Homo sapiens phosphatase and actin regulator 1 (PHACTR1), mRNA.	6.72	6.53	0.88	0.0014
NM_014773.2	KIAA0141	Homo sapiens KIAA0141 (KIAA0141), mRNA.	8.90	8.72	0.88	0.0416
NM_133436.1	ASNS	Homo sapiens asparagine synthetase (ASNS), transcript variant 1, mRNA.	7.19	7.00	0.88	0.0182
NM_182627.1	WDR53	Homo sapiens WD repeat domain 53 (WDR53), mRNA.	6.97	6.78	0.88	0.0121
NM_013283.3	MAT2B	Homo sapiens methionine adenosyltransferase II, beta (MAT2B), transcript variant 1, mRNA.	6.81	6.63	0.88	0.0291
NM_001277.2	CHKA	Homo sapiens choline kinase alpha (CHKA), transcript variant 1, mRNA.	7.88	7.70	0.88	0.0415
NM_001675.2	ATF4	Homo sapiens activating transcription factor 4 (tax-responsive enhancer element B67) (ATF4), transcript variant 1, mRNA.	12.07	11.89	0.88	0.0404
NM_080687.1	UPF3A	Homo sapiens UPF3 regulator of nonsense transcripts homolog A (yeast) (UPF3A), transcript variant 2, mRNA.	7.75	7.57	0.88	0.0423
NM_032529.1	KIAA1875	Homo sapiens KIAA1875 (KIAA1875), mRNA.	7.14	6.96	0.88	0.0279
NM_020650.2	RCN3	Homo sapiens reticulocalbin 3, EF-hand calcium binding domain (RCN3), mRNA.	7.55	7.37	0.88	0.0159
NM_080632.1	UPF3B	Homo sapiens UPF3 regulator of nonsense transcripts homolog B (yeast) (UPF3B), transcript variant 1, mRNA.	7.86	7.68	0.88	0.0262
NM_001031712.1	C6ORF75	Homo sapiens chromosome 6 open reading frame 75 (C6orf75), transcript variant 1, mRNA.	7.07	6.89	0.88	0.0335
NM_012411.2	PTPN22	Homo sapiens protein tyrosine phosphatase, non-receptor type 22 (lymphoid) (PTPN22), transcript variant 2, mRNA.	7.32	7.15	0.88	0.0369
NM_002084.2	GPX3	Homo sapiens glutathione peroxidase 3 (plasma) (GPX3), mRNA.	6.79	6.61	0.88	0.0170
NM_004075.2	CRY1	Homo sapiens cryptochrome 1 (photolyase-like) (CRY1), mRNA.	7.42	7.25	0.89	0.0156
NM_002600.2	PDE4B	Homo sapiens phosphodiesterase 4B, cAMP-specific (phosphodiesterase E4 dunce homolog, Drosophila) (PDE4B), mRNA.	6.77	6.59	0.89	0.0128
NM_012393.1	PFAS	Homo sapiens phosphoribosylformylglycinamidine synthase (FGAR amidotransferase) (PFAS), mRNA.	7.17	6.99	0.89	0.0415
NM_005690.2	DNM1L	Homo sapiens dynamin 1-like (DNM1L), transcript variant 3, mRNA.	7.33	7.16	0.89	0.0199
NM_015654.3	NAT9	Homo sapiens N-acetyltransferase 9 (NAT9), mRNA.	7.99	7.81	0.89	0.0489
NM_017736.3	THUMPD1	Homo sapiens THUMP domain containing 1 (THUMPD1), mRNA.	8.63	8.46	0.89	0.0314
NM_001033577.1	ZNHIT3	Homo sapiens zinc finger, HIT type 3 (ZNHIT3), transcript variant 1, mRNA.	8.36	8.19	0.89	0.0412
NM_002512.2	NME2	Homo sapiens non-metastatic cells 2, protein (NM23B) expressed in (NME2), transcript variant 1, mRNA.	7.13	6.97	0.89	0.0330
NM_003353.2	UCN	Homo sapiens urocortin (UCN), mRNA.	6.57	6.40	0.89	0.0042
NM_001039649.1	ZMYM5	Homo sapiens zinc finger, MYM-type 5 (ZMYM5), transcript variant 2, mRNA.	7.02	6.85	0.89	0.0119
NM_002182.2	IL1RAP	Homo sapiens interleukin 1 receptor accessory protein (IL1RAP), transcript variant 1, mRNA.	6.94	6.77	0.89	0.0168
NM_213603.2	LOC285989	Homo sapiens zinc finger protein 789 (ZNF789), transcript variant 1, mRNA.	7.33	7.17	0.89	0.0410
NM_024120.3	C20ORF7	Homo sapiens chromosome 20 open reading frame 7 (C20orf7), transcript variant 1, mRNA.	6.86	6.70	0.89	0.0152
NM_182646.1	CPEB2	Homo sapiens cytoplasmic polyadenylation element binding protein 2 (CPEB2), transcript variant A, mRNA.	7.81	7.65	0.89	0.0342
NM_052926.1	PNMA5	Homo sapiens paraneoplastic antigen like 5 (PNMA5), mRNA.	6.64	6.47	0.89	0.0077
NM_182686.1	KIAA0319L	Homo sapiens KIAA0319-like (KIAA0319L), transcript variant 2, mRNA.	7.02	6.86	0.90	0.0334
NM_020311.1	CMKOR1	Homo sapiens chemokine orphan receptor 1 (CMKOR1), mRNA.	6.61	6.46	0.90	0.0052
NM_018082.3	POLR3B	Homo sapiens polymerase (RNA) III (DNA directed) polypeptide B (POLR3B), mRNA.	7.24	7.08	0.90	0.0336
NM_025249.1	KIAA1683	Homo sapiens KIAA1683 (KIAA1683), mRNA.	6.68	6.52	0.90	0.0254
NM_006372.3	SYNCRIP	Homo sapiens synaptotagmin binding, cytoplasmic RNA interacting protein (SYNCRIP), mRNA.	8.79	8.64	0.90	0.0448
NM_018590.3	GALNACT-2	Homo sapiens chondroitin sulfate GalNAcT-2 (GALNACT-2), mRNA.	6.93	6.77	0.90	0.0395

NM_016389.2	IVNS1ABP	Homo sapiens influenza virus NS1A binding protein (IVNS1ABP), transcript variant 2, mRNA.	6.71	6.55	0.90	0.0263
NM_025000.1	FLJ13096	Homo sapiens hypothetical protein FLJ13096 (FLJ13096), mRNA.	6.48	6.32	0.90	0.0017
NM_006575.3	MAP4K5	Homo sapiens mitogen-activated protein kinase kinase kinase kinase 5 (MAP4K5), transcript variant 1, mRNA.	6.48	6.33	0.90	0.0134
NM_001008401.1	FLJ16231	Homo sapiens FLJ16231 protein (FLJ16231), mRNA.	6.55	6.40	0.90	0.0199
NM_015326.2	SRGAP2	Homo sapiens SLIT-ROBO Rho GTPase activating protein 2 (SRGAP2), transcript variant 1, mRNA.	6.70	6.55	0.90	0.0249
NM_013364.2	PNMA3	Homo sapiens paraneoplastic antigen MA3 (PNMA3), mRNA.	6.48	6.33	0.90	0.0098
NM_181701.2	QSCN6L1	Homo sapiens quiescin Q6-like 1 (QSCN6L1), mRNA.	7.30	7.15	0.90	0.0423
NM_001292.1	CLK3	Homo sapiens CDC-like kinase 3 (CLK3), transcript variant phclk3/152, mRNA.	6.69	6.54	0.90	0.0128
NM_007362.2	NCBP2	Homo sapiens nuclear cap binding protein subunit 2, 20kDa (NCBP2), mRNA.	7.55	7.40	0.90	0.0485
NM_015387.2	PREI3	Homo sapiens preimplantation protein 3 (PREI3), transcript variant 1, mRNA.	6.54	6.39	0.90	0.0023
NM_004733.2	SLC33A1	Homo sapiens solute carrier family 33 (acetyl-CoA transporter), member 1 (SLC33A1), mRNA.	6.77	6.62	0.90	0.0354
NM_001229.2	CASP9	Homo sapiens caspase 9, apoptosis-related cysteine peptidase (CASP9), transcript variant alpha, mRNA.	6.53	6.38	0.91	0.0027
NM_001029840.1	C3ORF23	Homo sapiens chromosome 3 open reading frame 23 (C3orf23), transcript variant 3, mRNA.	6.60	6.46	0.91	0.0051
NM_001907.1	CTRL	Homo sapiens chymotrypsin-like (CTRL), mRNA.	7.13	6.98	0.91	0.0295
NM_002114.1	HIVEP1	Homo sapiens human immunodeficiency virus type I enhancer binding protein 1 (HIVEP1), mRNA.	7.07	6.93	0.91	0.0377
NM_016437.1	TUBG2	Homo sapiens tubulin, gamma 2 (TUBG2), mRNA.	7.20	7.06	0.91	0.0276
NM_014970.2	KIFAP3	Homo sapiens kinesin-associated protein 3 (KIFAP3), mRNA.	6.98	6.84	0.91	0.0289
NM_000056.2	BCKDHB	Homo sapiens branched chain keto acid dehydrogenase E1, beta polypeptide (maple syrup urine disease) (BCKDHB), nuclear gene encoding mitoch	6.53	6.40	0.91	0.0024
NM_018991.2	DKFZP434A0131	Homo sapiens DKFZp434A0131 protein (DKFZP434A0131), transcript variant 1, mRNA.	6.45	6.31	0.91	0.0101
NM_030971.3	SFXN3	Homo sapiens sideroflexin 3 (SFXN3), mRNA.	6.50	6.37	0.91	0.0027
NM_182926.1	KTN1	Homo sapiens kinectin 1 (kinesin receptor) (KTN1), mRNA.	6.81	6.67	0.91	0.0353
NM_007045.2	FGFR1OP	Homo sapiens FGFR1 oncogene partner (FGFR1OP), transcript variant 1, mRNA.	6.84	6.70	0.91	0.0421
NR_002448.1	SNORD36A	Homo sapiens small nucleolar RNA, C/D box 36A (SNORD36A) on chromosome 9.	6.68	6.55	0.91	0.0055
NM_172107.1	KCNQ2	Homo sapiens potassium voltage-gated channel, KQT-like subfamily, member 2 (KCNQ2), transcript variant 1, mRNA.	6.62	6.49	0.91	0.0230
NM_015419.1	MXRA5	Homo sapiens matrix-remodelling associated 5 (MXRA5), mRNA.	6.64	6.50	0.91	0.0135
NM_198460.1	GBP6	Homo sapiens guanylate binding protein family, member 6 (GBP6), mRNA.	6.47	6.34	0.91	0.0121
NM_001032282.1	KLF10	Homo sapiens Kruppel-like factor 10 (KLF10), transcript variant 2, mRNA.	6.73	6.60	0.91	0.0340
NM_033402.2	LRRCC1	Homo sapiens leucine rich repeat and coiled-coil domain containing 1 (LRRCC1), mRNA.	6.53	6.40	0.91	0.0043
NM_005698.2	SCAMP3	Homo sapiens secretory carrier membrane protein 3 (SCAMP3), transcript variant 1, mRNA.	6.64	6.51	0.91	0.0182
NM_023000.2	ARID4A	Homo sapiens AT rich interactive domain 4A (RBP1-like) (ARID4A), transcript variant 2, mRNA.	6.73	6.60	0.91	0.0327
NM_002814.2	PSMD10	Homo sapiens proteasome (prosome, macropain) 26S subunit, non-ATPase, 10 (PSMD10), transcript variant 1, mRNA.	6.89	6.77	0.92	0.0482
NM_001951.2	E2F5	Homo sapiens E2F transcription factor 5, p130-binding (E2F5), mRNA.	6.58	6.45	0.92	0.0086
NM_152221.2	CSNK1E	Homo sapiens casein kinase 1, epsilon (CSNK1E), transcript variant 1, mRNA.	6.59	6.46	0.92	0.0167
NM_007214.3	SEC63	Homo sapiens SEC63 homolog (S. cerevisiae) (SEC63), mRNA.	6.64	6.52	0.92	0.0128
NM_003035.2	STIL	Homo sapiens SCL/TAL1 interrupting locus (STIL), transcript variant 2, mRNA.	6.63	6.51	0.92	0.0252
NM_006996.1	SLC19A2	Homo sapiens solute carrier family 19 (thiamine transporter), member 2 (SLC19A2), mRNA.	6.50	6.37	0.92	0.0045
NM_016488.5	PPHLN1	Homo sapiens periphilin 1 (PPHLN1), transcript variant 1, mRNA.	6.78	6.66	0.92	0.0297
NM_052963.1	TOP1MT	Homo sapiens topoisomerase (DNA) I, mitochondrial (TOP1MT), nuclear gene encoding mitochondrial protein, mRNA.	6.89	6.76	0.92	0.0492
NM_030965.1	ST6GALNAC5	Homo sapiens ST6 (alpha-N-acetyl-neuraminyl-2,3-beta-galactosyl-1, 3)-N-acetylgalactosaminide alpha-2,6-sialyltransferase 5 (ST6GALNAC5), mRN	6.56	6.44	0.92	0.0242
NM_014245.2	RNF7	Homo sapiens ring finger protein 7 (RNF7), transcript variant 1, mRNA.	6.55	6.42	0.92	0.0209
NM_006080.1	SEMA3A	Homo sapiens sema domain, immunoglobulin domain (Ig), short basic domain, secreted, (semaphorin) 3A (SEMA3A), mRNA.	6.66	6.54	0.92	0.0138
NM_017984.2	ZCWPW1	Homo sapiens zinc finger, CW type with PWWP domain 1 (ZCWPW1), mRNA.	6.65	6.53	0.92	0.0206
NM_006813.1	PNRC1	Homo sapiens proline-rich nuclear receptor coactivator 1 (PNRC1), mRNA.	6.63	6.52	0.92	0.0229
NM_015316.1	PPP1R13B	Homo sapiens protein phosphatase 1, regulatory (inhibitor) subunit 13B (PPP1R13B), mRNA.	6.65	6.53	0.92	0.0331
NM_006205.1	PDE6H	Homo sapiens phosphodiesterase 6H, cGMP-specific, cone, gamma (PDE6H), mRNA.	6.51	6.39	0.92	0.0199
NM_032207.1	FLJ21742	Homo sapiens hypothetical protein FLJ21742 (FLJ21742), mRNA.	6.71	6.60	0.92	0.0389
NM_005371.3	METTL1	Homo sapiens methyltransferase like 1 (METTL1), transcript variant 1, mRNA.	6.62	6.51	0.93	0.0457
NM_021777.2	ADAM28	Homo sapiens ADAM metallopeptidase domain 28 (ADAM28), transcript variant 3, mRNA.	6.51	6.40	0.93	0.0370
NM_198149.1	TMEM58	Homo sapiens transmembrane protein 58 (TMEM58), mRNA.	6.49	6.38	0.93	0.0095
NM_182966.1	NEDD9	Homo sapiens neural precursor cell expressed, developmentally down-regulated 9 (NEDD9), transcript variant 2, mRNA.	6.51	6.40	0.93	0.0495
NM_001042610.1	DBNDD1	Homo sapiens dysbindin (dystrobrevin binding protein 1) domain containing 1 (DBNDD1), transcript variant 1, mRNA.	6.60	6.49	0.93	0.0358
NM_080680.1	COL11A2	Homo sapiens collagen, type XI, alpha 2 (COL11A2), transcript variant 1, mRNA.	6.58	6.47	0.93	0.0306
NM_005995.2	TBX10	Homo sapiens T-box 10 (TBX10), mRNA.	6.55	6.44	0.93	0.0438
NM_000864.3	HTR1D	Homo sapiens 5-hydroxytryptamine (serotonin) receptor 1D (HTR1D), mRNA.	6.53	6.42	0.93	0.0213
NM_203411.1	TMEM88	Homo sapiens transmembrane protein 88 (TMEM88), mRNA.	6.61	6.51	0.93	0.0347
NM_173803.2	FLJ39599	Homo sapiens MPV17 mitochondrial membrane protein-like (MPV17L), mRNA.	6.59	6.49	0.93	0.0464
NM_001010870.1	TDRD6	Homo sapiens tudor domain containing 6 (TDRD6), mRNA.	6.63	6.53	0.93	0.0392
NM_021777.2	ADAM28	Homo sapiens ADAM metallopeptidase domain 28 (ADAM28), transcript variant 3, mRNA.	6.45	6.35	0.94	0.0362
NM_001018116.1	LOC347273	Homo sapiens similar to RIKEN cDNA 2310039E09 (LOC347273), mRNA.	6.53	6.44	0.94	0.0159
NM_153605.2	DKFZP667G2110	Homo sapiens hypothetical protein DKFZp667G2110 (DKFZp667G2110), mRNA.	6.46	6.37	0.94	0.0461
NM_001009957.1	ZNF655	Homo sapiens zinc finger protein 655 (ZNF655), transcript variant 5, mRNA.	6.51	6.42	0.94	0.0493
NM_001080829.1	LOC390688	Homo sapiens CDC37-like (LOC390688), mRNA.	6.46	6.37	0.94	0.0454
NM_033118.2	MYLK2	Homo sapiens myosin light chain kinase 2, skeletal muscle (MYLK2), mRNA.	6.44	6.36	0.94	0.0428
XM_926644.1	LOC643298	PREDICTED: Homo sapiens similar to Thyroid hormone receptor-associated protein complex 240 kDa component (Trap240) (Thyroid hormone recep	6.49	6.41	0.94	0.0485
