# Supplementary material for: Short-Term Erythropoietin Treatment Does Not Substantially Modulate Monocyte Transcriptomes of Patients with Combined Heart and Renal Failure
Source: PLoS One. 2012 Sep 5;7(9):e41339. doi: 10.1371/journal.pone.0041339 (PMC3434212; doi:10.1371/journal.pone.0041339)
Supplement: Table S2 — Cardiorenal connector monocyte gene expression panel: differences in CRS patients at baseline (n = 18) compared to healthy controls (n = 12). (PDF) [file pone.0041339.s003.pdf]

Supplementary material table 2: Cardiorenal connector monocyte gene expression panel: differences in CRS patients at baseline (n=18) compared to healthy controls (n=12)

Inflammation related genes

Symbol	Fold change	P-value
IL10	0.91	0.369
IL10RA	0.91	0.222
IL10RB	1.15	0.044
IL12A	not expressed	not expressed
IL12RB1	1.10	0.006
IL13RA1	1.15	0.037
IL17C	1.03	0.471
IL17D	1.04	0.404
IL17E	not expressed	not expressed
IL17R	1.19	0.012
IL1B	0.82	0.080
IL1F7	0.96	0.664
IL1F8	not expressed	not expressed
IL1R2	0.83	0.044
IL1RAP	0.89	0.017
IL1RL1	0.97	0.382
IL1RN	0.94	0.052
IL21	not expressed	not expressed
IL21R	1.10	0.066
IL27	1.03	0.472
IL27RA	1.22	0.001
IL2RG	not expressed	not expressed
IL4I1	not expressed	not expressed
IL4R	1.12	0.151
IL5RA	1.03	0.587
IL6	not expressed	not expressed
IL6R	0.92	0.317
IL8	0.58	0.001
IL8RB	0.87	0.079
IFNA10	1.06	0.577
IFNA14	not expressed	not expressed
IFNA21	1.01	0.824
IFNA8	1.00	0.945
IFNAR1	1.06	0.530
IFNAR2	1.07	0.410
IFNGR1	1.10	0.123
IFNGR2	1.08	0.298
TNF	1.06	0.360
CCL19	0.97	0.419
CCL2	0.77	0.008
CCL21	not expressed	not expressed
CCL23	1.02	0.618
CCL25	1.03	0.368
CCL26	0.99	0.813
CCL27	not expressed	not expressed
CCL28	not expressed	not expressed

Symbol	Fold change	P-value
CCL3	0.86	0.027
CCL3L1	not expressed	not expressed
CCL3L3	0.77	0.019
CCL4L1	not expressed	not expressed
CCL5	0.98	0.870
CCL8	0.89	0.097
CXCL10	0.94	0.506
CXCL12	not expressed	not expressed
CXCL14	0.98	0.680
CXCL16	0.90	0.218
CXCL9	not expressed	not expressed
CXCR4	0.90	0.221
CCR1	1.09	0.206
CCR10	not expressed	not expressed
CCR2	1.11	0.274
CCR6	1.02	0.791
CCR7	not expressed	not expressed
CX3CR1	1.49	0.0002
CCXCR1	not expressed	not expressed
TLR1	1.05	0.615
TLR10	not expressed	not expressed
TLR2	1.16	0.015
TLR4	0.99	0.864
TLR5	0.97	0.741
TLR6	1.09	0.021
TLR7	1.20	0.040
TLR8	1.03	0.407
TLR9	not expressed	not expressed
IRF1	1.00	0.988
IRF3	1.04	0.536
IRF4	1.02	0.798
IRF5	1.44	0.051
IRF7	1.09	0.359
IRF8	1.11	0.136

Oxidative stress related genes

Symbol	Fold change	P-value
GPX1	0.93	0.119
GPX1	1.12	0.175
GPX2	1.02	0.470
GPX3	0.88	0.017
GPX4	0.98	0.747
GPX4	1.01	0.940
GPX6	1.01	0.741
GPX7	0.95	0.380
PRDX1	0.98	0.756
PRDX1	0.98	0.862
PRDX2	0.89	0.167
PRDX3	0.85	0.061
PRDX4	1.04	0.548
PRDX5	0.91	0.277
PRDX5	0.98	0.776
PRDX5	1.02	0.779
PRDX6	0.99	0.916
TXNRD1	1.05	0.390
TXNRD1	0.99	0.901
TXNRD2	1.04	0.618
SRXN1	1.01	0.897
SELS	0.94	0.060
DHRS9	1.33	0.006
GSR	0.97	0.356
SOD1	1.02	0.800
SOD2	0.93	0.213
SOD2	0.95	0.582
SOD2	0.99	0.901
CCS	0.92	0.182
CCS	1.06	0.356
CYBA	1.08	0.167
DUOX1	1.01	0.752
NCF1	0.93	0.376
NCF2	1.00	0.964
NOS3	0.98	0.372
NOX4	0.99	0.719
NOX5	1.05	0.052
PREX1	1.09	0.212
ALOX15	0.98	0.458
ALOX15B	0.93	0.084
ALOX15B	0.97	0.401
ALOX15B	0.99	0.832
ALOX5	1.03	0.322
ALOX5	1.04	0.634
ALOX5AP	1.11	0.283
ALOXE3	1.01	0.741
DUSP1	0.68	0.003
MPO	0.92	0.175

Renin angiotensin system related genes

Symbol	Fold change	P-value
AGTR1	not expressed	not expressed
AGT	not expressed	not expressed
REN	0.99	0.809
ACE	not expressed	not expressed
RENBP	1.18	0.052
