# Supplementary material for: Short-Term Erythropoietin Treatment Does Not Substantially Modulate Monocyte Transcriptomes of Patients with Combined Heart and Renal Failure
Source: PLoS One. 2012 Sep 5;7(9):e41339. doi: 10.1371/journal.pone.0041339 (PMC3434212; doi:10.1371/journal.pone.0041339)
Supplement: Table S3 — Monocyte gene expression changes in CRS patients (n = 12) after 2 weeks of EPO treatment. (PDF) [file pone.0041339.s004.pdf]

Target ID	Symbol	Annotation	Fold change	p
3440739	HBB	Homo sapiens hemoglobin, beta (HBB), mRNA.	3.49	0.007
7570408	CCL5	Homo sapiens chemokine (C-C motif) ligand 5 (CCL5), mRNA.	1.26	0.042
7040132	ANKRD12	Homo sapiens ankyrin repeat domain 12 (ANKRD12), mRNA.	1.19	0.005
3870338	IFI44L	Homo sapiens interferon-induced protein 44-like (IFI44L), mRNA.	1.18	0.046
1410537	RPSA	Homo sapiens ribosomal protein SA (RPSA), transcript variant 1, mRNA.	1.18	0.002
1050082	KIAA1147	Homo sapiens KIAA1147 (KIAA1147), mRNA.	1.17	0.041
5080605	SNRPA1	Homo sapiens small nuclear ribonucleoprotein polypeptide A' (SNRPA1), mRNA.	1.16	0.030
1510091	VPS36	Homo sapiens vacuolar protein sorting 36 homolog (S. cerevisiae) (VPS36), mRNA.	1.16	0.012
540390	ALPK1	Homo sapiens alpha-kinase 1 (ALPK1), mRNA.	1.16	0.002
7610440	XAF1	Homo sapiens XIAP associated factor-1 (XAF1), transcript variant 2, mRNA.	1.16	0.024
1820037	SLC11A2	Homo sapiens solute carrier family 11 (proton-coupled divalent metal ion transporters), member 2 (SLC1	1.16	0.018
4860064	DDX19B	Homo sapiens DEAD (Asp-Glu-Ala-As) box polypeptide 19B (DDX19B), transcript variant 2, mRNA.	1.16	0.005
5720482	HERC5	Homo sapiens hect domain and RLD 5 (HERC5), mRNA.	1.16	0.038
6290379	TMEM29	Homo sapiens transmembrane protein 29 (TMEM29), mRNA.	1.16	0.013
4810390	TROVE2	Homo sapiens TROVE domain family, member 2 (TROVE2), transcript variant 1, mRNA.	1.15	0.043
1580035	VPS26A	Homo sapiens vacuolar protein sorting 26 homolog A (S. pombe) (VPS26A), transcript variant 2, mRNA.	1.15	0.024
5960747	TRIM22	Homo sapiens tripartite motif-containing 22 (TRIM22), mRNA.	1.15	0.036
5890047	ZDHHC17	Homo sapiens zinc finger, DHHC-type containing 17 (ZDHHC17), mRNA.	1.15	0.029
3870619	AMD1	Homo sapiens adenosylmethionine decarboxylase 1 (AMD1), transcript variant 1, mRNA.	1.15	0.006
5870180	ZNF277	Homo sapiens zinc finger protein 277 (ZNF277), mRNA.	1.15	0.032
580184	PHF20L1	Homo sapiens PHD finger protein 20-like 1 (PHF20L1), transcript variant 1, mRNA.	1.15	0.028
1710369	RPL3	Homo sapiens ribosomal protein L3 (RPL3), transcript variant 2, mRNA.	1.14	0.014
6510546	PCNP	Homo sapiens PEST proteolytic signal containing nuclear protein (PCNP), mRNA.	1.14	0.019
5810022	MLKL	Homo sapiens mixed lineage kinase domain-like (MLKL), mRNA.	1.14	0.033
1010168	DSCR2	Homo sapiens Down syndrome critical region gene 2 (DSCR2), transcript variant 2, mRNA.	1.14	0.015
580242	LRDD	Homo sapiens leucine-rich repeats and death domain containing (LRDD), transcript variant 2, mRNA.	1.14	0.002
6660377	THOC2	Homo sapiens THO complex 2 (THOC2), mRNA.	1.14	0.019
5960014	CCDC32	Homo sapiens coiled-coil domain containing 32 (CCDC32), transcript variant 2, mRNA.	1.14	0.020
2120376	C6ORF111	Homo sapiens chromosome 6 open reading frame 111 (C6orf111), mRNA.	1.13	0.047
4070289	FLJ10006	Homo sapiens IWS1 homolog (S. cerevisiae) (IWS1), mRNA.	1.13	0.006
6350332	SEL1L	Homo sapiens sel-1 suppressor of lin-12-like (C. elegans) (SEL1L), mRNA.	1.13	0.002
4900209	TIMM9	Homo sapiens translocase of inner mitochondrial membrane 9 homolog (yeast) (TIMM9), mRNA.	1.13	0.021
6480403	TXNDC3	Homo sapiens thioredoxin domain containing 3 (spermatozoa) (TXNDC3), mRNA.	1.13	0.018
10253	PPIL5	Homo sapiens peptidylprolyl isomerase (cyclophilin)-like 5 (PPIL5), transcript variant 1, mRNA.	1.13	0.008
2350468	SENP7	Homo sapiens SUMO1/sentrin specific peptidase 7 (SENP7), transcript variant 2, mRNA.	1.12	0.008
2850059	CRY1	Homo sapiens cryptochrome 1 (photolyase-like) (CRY1), mRNA.	1.12	0.023
3940451	PSRC2	Homo sapiens proline/serine-rich coiled-coil 2 (PSRC2), mRNA.	1.12	0.025
1070300	OGT	Homo sapiens O-linked N-acetylglucosamine (GlcNAc) transferase (UDP-N-acetylglucosamine:polypept	1.12	0.049
2510278	MAD2L2	Homo sapiens MAD2 mitotic arrest deficient-like 2 (yeast) (MAD2L2), mRNA.	1.12	0.036
6100441	PRPF4	Homo sapiens PRP4 pre-mRNA processing factor 4 homolog (yeast) (PRPF4), mRNA.	1.12	0.028
4070367	NSUN5B	Homo sapiens NOL1/NOP2/Sun domain family, member 5B (NSUN5B), transcript variant 2, mRNA.	1.12	0.014
2370601	FLJ10374	Homo sapiens hypothetical protein FLJ10374 (FLJ10374), mRNA.	1.12	0.017
6590484	NAP1L1	Homo sapiens nucleosome assembly protein 1-like 1 (NAP1L1), transcript variant 1, mRNA.	1.12	0.037
5550431	MMD	Homo sapiens monocyte to macrophage differentiation-associated (MMD), mRNA.	1.12	0.009
20427	RNH1	Homo sapiens ribonuclease/angiogenin inhibitor 1 (RNH1), transcript variant 4, mRNA.	1.12	0.030
5810630	DSCR5	Homo sapiens Down syndrome critical region gene 5 (DSCR5), transcript variant 1, mRNA.	1.12	0.032
2340484	SCD5	Homo sapiens stearoyl-CoA desaturase 5 (SCD5), transcript variant 2, mRNA.	1.12	0.014
780753	SUPT16H	Homo sapiens suppressor of Ty 16 homolog (S. cerevisiae) (SUPT16H), mRNA.	1.11	0.047
430142	HSPA4	Homo sapiens heat shock 70kDa protein 4 (HSPA4), transcript variant 1, mRNA.	1.11	0.017
4210470	C14ORF135	Homo sapiens chromosome 14 open reading frame 135 (C14orf135), mRNA.	1.11	0.020
5860204	FKBP11	Homo sapiens FK506 binding protein 11, 19 kDa (FKBP11), mRNA.	1.11	0.021
7050139	DKFZP586I14	Homo sapiens hypothetical protein DKFZp586I1420 (DKFZp586I1420) on chromosome 7.	1.11	0.027
60184	NDN	Homo sapiens necdin homolog (mouse) (NDN), mRNA.	1.11	0.041
3060458	FAM111A	Homo sapiens family with sequence similarity 111, member A (FAM111A), transcript variant 1, mRNA.	1.11	0.036
7040717	AASDHPPT	Homo sapiens aminoadipate-semialdehyde dehydrogenase-phosphopantetheinyl transferase (AASDHP	1.11	0.039
2470762	KIAA0256	Homo sapiens KIAA0256 gene product (KIAA0256), mRNA.	1.11	0.019
5690181	KIAA1712	Homo sapiens KIAA1712 (KIAA1712), mRNA.	1.11	0.045
7320424	HNRPA1L-2	Homo sapiens heterogeneous nuclear ribonucleoprotein A1 pseudogene (HNRPA1L-2) on chromosome	1.11	0.031
6370113	ALG5	Homo sapiens asparagine-linked glycosylation 5 homolog (S. cerevisiae, dolichyl-phosphate beta-glucos	1.11	0.042
6060646	CRYZL1	Homo sapiens crystallin, zeta (quinone reductase)-like 1 (CRYZL1), transcript variant 1, mRNA.	1.11	0.043
7510551	FLJ21986	Homo sapiens hypothetical protein FLJ21986 (FLJ21986), mRNA.	1.11	0.029
2350068	WDR12	Homo sapiens WD repeat domain 12 (WDR12), mRNA.	1.11	0.030
1710026	MGC39633	Homo sapiens coiled-coil domain containing 112 (CCDC112), transcript variant 2, mRNA.	1.11	0.028
510471	KIAA0157	Homo sapiens KIAA0157 (KIAA0157), mRNA.	1.11	0.049
520196	GNL2	Homo sapiens guanine nucleotide binding protein-like 2 (nucleolar) (GNL2), mRNA.	1.11	0.044
10386	TM2D1	Homo sapiens TM2 domain containing 1 (TM2D1), mRNA.	1.10	0.045
1820360	MAPK9	Homo sapiens mitogen-activated protein kinase 9 (MAPK9), transcript variant JNK2-a2, mRNA.	1.10	0.024

Target ID	Symbol	Annotation	Fold change	p
2350541	VAMP1	Homo sapiens vesicle-associated membrane protein 1 (synaptobrevin 1) (VAMP1), transcript variant 1, r	1.10	0.026
3850121	EEF1A1	Homo sapiens eukaryotic translation elongation factor 1 alpha 1 (EEF1A1), mRNA.	1.10	0.048
1940576	RPS6KB1	Homo sapiens ribosomal protein S6 kinase, 70kDa, polypeptide 1 (RPS6KB1), mRNA.	1.10	0.037
5910594	PTP4A3	Homo sapiens protein tyrosine phosphatase type IVA, member 3 (PTP4A3), transcript variant 2, mRNA.	1.10	0.032
6480053	ATF4	Homo sapiens activating transcription factor 4 (tax-responsive enhancer element B67) (ATF4), transcript	1.10	0.006
6860347	FAM46C	Homo sapiens family with sequence similarity 46, member C (FAM46C), mRNA.	1.10	0.025
460259	LAT	Homo sapiens linker for activation of T cells (LAT), transcript variant 2, mRNA.	1.10	0.007
3930286	SUCLA2	Homo sapiens succinate-CoA ligase, ADP-forming, beta subunit (SUCLA2), mRNA.	1.10	0.032
2360561	TMEM70	Homo sapiens transmembrane protein 70 (TMEM70), transcript variant 1, mRNA.	1.10	0.006
3890500	MRPS21	Homo sapiens mitochondrial ribosomal protein S21 (MRPS21), nuclear gene encoding mitochondrial pro	1.10	0.033
7570240	GLMN	Homo sapiens glomulin, FKBP associated protein (GLMN), transcript variant 1, mRNA.	1.10	0.017
2340753	TNFRSF13C	Homo sapiens tumor necrosis factor receptor superfamily, member 13C (TNFRSF13C), mRNA.	1.10	0.030
1070450	PTGS1	Homo sapiens prostaglandin-endoperoxide synthase 1 (prostaglandin G/H synthase and cyclooxygenas	1.10	0.034
1030201	WDR33	Homo sapiens WD repeat domain 33 (WDR33), transcript variant 3, mRNA.	1.10	0.042
6250100	CSNK1E	Homo sapiens casein kinase 1, epsilon (CSNK1E), transcript variant 1, mRNA.	1.10	0.009
4230053	GPATC3	Homo sapiens G patch domain containing 3 (GPATCH3), mRNA.	1.10	0.023
6770360	39509	PREDICTED: Homo sapiens membrane-associated ring finger (C3HC4) 2 (MARCH2), mRNA.	1.10	0.016
6060379	VPS54	Homo sapiens vacuolar protein sorting 54 homolog (S. cerevisiae) (VPS54), transcript variant 1, mRNA.	1.10	0.024
1580544	PIGH	Homo sapiens phosphatidylinositol glycan, class H (PIGH), mRNA.	1.10	0.025
3290170	WDR71	Homo sapiens WD repeat domain 71 (WDR71), mRNA.	1.10	0.032
270204	RFNG	PREDICTED: Homo sapiens radical fringe homolog (Drosophila) (RFNG), mRNA.	1.10	0.025
6060739	ZNF451	Homo sapiens zinc finger protein 451 (ZNF451), transcript variant 1, mRNA.	1.09	0.022
1410307			1.09	0.006
130647	PEX1	Homo sapiens peroxisome biogenesis factor 1 (PEX1), mRNA.	1.09	0.032
7000672	AGL	Homo sapiens amylo-1, 6-glucosidase, 4-alpha-glucanotransferase (glycogen debranching enzyme, glyc	1.09	0.008
2940070	GOLGA4	Homo sapiens golgi autoantigen, golgin subfamily a, 4 (GOLGA4), mRNA.	1.09	0.011
6660195	RPUSD3	Homo sapiens RNA pseudouridylate synthase domain containing 3 (RPUSD3), mRNA.	1.09	0.029
5720162	ANKRD26	Homo sapiens ankyrin repeat domain 26 (ANKRD26), mRNA.	1.09	0.020
5390010	SLC9A8	Homo sapiens solute carrier family 9 (sodium/hydrogen exchanger), member 8 (SLC9A8), mRNA.	1.09	0.033
6420184	DENND4A	Homo sapiens DENN/MADD domain containing 4A (DENND4A), mRNA.	1.09	0.046
2450162	SFTPG	Homo sapiens surfactant associated protein G (SFTPG), mRNA.	1.09	0.023
430731	WDR53	Homo sapiens WD repeat domain 53 (WDR53), mRNA.	1.09	0.023
1980338	C6ORF84	Homo sapiens KIAA1009 (KIAA1009), mRNA.	1.09	0.018
4050427	IHPK3	Homo sapiens inositol hexaphosphate kinase 3 (IHPK3), mRNA.	1.09	0.019
2650092	JOSD2	Homo sapiens Josephin domain containing 2 (JOSD2), mRNA.	1.09	0.019
2650494	SMC2L1	Homo sapiens SMC2 structural maintenance of chromosomes 2-like 1 (yeast) (SMC2L1), mRNA.	1.09	0.012
4760725	PRPF38B	Homo sapiens PRP38 pre-mRNA processing factor 38 (yeast) domain containing B (PRPF38B), mRNA.	1.09	0.024
6580653	UBE1DC1	Homo sapiens ubiquitin-activating enzyme E1-domain containing 1 (UBE1DC1), transcript variant 1, mR	1.09	0.044
430408	DPP3	Homo sapiens dipeptidylpeptidase 3 (DPP3), transcript variant 2, mRNA.	1.08	0.017
7560452	MULK	Homo sapiens acylglycerol kinase (AGK), mRNA.	1.08	0.039
6370273	CTPS	Homo sapiens CTP synthase (CTPS), mRNA.	1.08	0.042
1980192	INVS	Homo sapiens inversin (INVS), transcript variant 2, mRNA.	1.08	0.036
2570605	ARHGAP5	Homo sapiens Rho GTPase activating protein 5 (ARHGAP5), transcript variant 2, mRNA.	1.08	0.023
5080162	KTN1	Homo sapiens kinectin 1 (kinesin receptor) (KTN1), mRNA.	1.08	0.021
5910274	KBTBD3	Homo sapiens kelch repeat and BTB (POZ) domain containing 3 (KBTBD3), transcript variant 2, mRNA.	1.08	0.027
5890139	CPXM2	Homo sapiens carboxypeptidase X (M14 family), member 2 (CPXM2), mRNA.	1.08	0.042
6550632	ZNF219	Homo sapiens zinc finger protein 219 (ZNF219), mRNA.	1.08	0.034
5810221	CACNA2D4	Homo sapiens calcium channel, voltage-dependent, alpha 2/delta subunit 4 (CACNA2D4), transcript var	1.08	0.049
7510026	BRF1	Homo sapiens BRF1 homolog, subunit of RNA polymerase III transcription initiation factor IIIB (S. cerevi	1.08	0.015
2750392	EPM2A	Homo sapiens epilepsy, progressive myoclonus type 2A, Lafora disease (laforin) (EPM2A), transcript va	1.08	0.010
4850725	PSEN1	Homo sapiens presenilin 1 (Alzheimer disease 3) (PSEN1), mRNA.	1.08	0.042
3850092	LMAN2	Homo sapiens lectin, mannose-binding 2 (LMAN2), mRNA.	1.08	0.028
380348	C9ORF82	Homo sapiens chromosome 9 open reading frame 82 (C9orf82), mRNA.	1.08	0.045
3130753	GCNT2	Homo sapiens glucosaminyl (N-acetyl) transferase 2, I-branching enzyme (GCNT2), transcript variant 1,	1.08	0.047
5550170	ARHGEF10L	Homo sapiens Rho guanine nucleotide exchange factor (GEF) 10-like (ARHGEF10L), transcript variant	1.08	0.040
940463	PNPLA1	Homo sapiens patatin-like phospholipase domain containing 1 (PNPLA1), transcript variant 2, mRNA.	1.08	0.012
1500463	ZNF37A	Homo sapiens zinc finger protein 37A (ZNF37A), transcript variant 1, mRNA.	1.08	0.024
6560131	KIAA1411	Homo sapiens family with sequence similarity 135, member A (FAM135A), mRNA.	1.08	0.049
870646	SPFH2	Homo sapiens ER lipid raft associated 2 (ERLIN2), transcript variant 2, mRNA.	1.08	0.018
650372	UBE2Z	PREDICTED: Homo sapiens ubiquitin-conjugating enzyme E2Z (putative), transcript variant 3 (UBE2Z),	1.08	0.040
4120754	DCUN1D4	Homo sapiens DCN1, defective in cullin neddylation 1, domain containing 4 (S. cerevisiae) (DCUN1D4),	1.07	0.045
4200019	CTNND1	PREDICTED: Homo sapiens catenin (cadherin-associated protein), delta 1, transcript variant 5 (CTNND	1.07	0.048
1580014	PLD4	Homo sapiens phospholipase D family, member 4 (PLD4), mRNA.	1.07	0.010
1010139	LHFP	Homo sapiens lipoma HMGIC fusion partner (LHFP), mRNA.	1.07	0.022
1170154	ZNF3	Homo sapiens zinc finger protein 3 (ZNF3), transcript variant 1, mRNA.	1.07	0.035
430376	ASMTL	Homo sapiens acetylserotonin O-methyltransferase-like (ASMTL), mRNA.	1.07	0.032
4860609	NEK9	Homo sapiens NIMA (never in mitosis gene a)- related kinase 9 (NEK9), mRNA.	1.07	0.017

Target ID	Symbol	Annotation	Fold change	p
2100563	QTRT1	Homo sapiens queuine tRNA-ribosyltransferase 1 (tRNA-guanine transglycosylase) (QTRT1), mRNA.	1.07	0.035
2100544	ZNF136	PREDICTED: Homo sapiens zinc finger protein 136 (clone pHZ-20) (ZNF136), mRNA.	1.07	0.034
5490343	GOLGA7	Homo sapiens golgi autoantigen, golgin subfamily a, 7 (GOLGA7), transcript variant 2, mRNA.	1.07	0.040
3930040	CRIM1	Homo sapiens cysteine rich transmembrane BMP regulator 1 (chordin-like) (CRIM1), mRNA.	1.07	0.017
1440615	OTOF	Homo sapiens otoferlin (OTOF), transcript variant 1, mRNA.	1.07	0.047
5290600	TTLL1	Homo sapiens tubulin tyrosine ligase-like family, member 1 (TTLL1), transcript variant 1, mRNA.	1.07	0.030
2370162	MEIS3	Homo sapiens Meis homeobox 3 (MEIS3), transcript variant 2, mRNA.	1.07	0.028
2970746	MGC24975	Homo sapiens hypothetical protein MGC24975 (MGC24975), mRNA.	1.07	0.047
4220215	DYNC2H1	Homo sapiens dynein, cytoplasmic 2, heavy chain 1 (DYNC2H1), mRNA.	1.07	0.017
4810167	C6ORF25	Homo sapiens chromosome 6 open reading frame 25 (C6orf25), transcript variant 4, mRNA.	1.06	0.045
5960070	ELAVL3	Homo sapiens ELAV (embryonic lethal, abnormal vision, Drosophila)-like 3 (Hu antigen C) (ELAVL3), tra	1.06	0.049
4390754	PPL	Homo sapiens periplakin (PPL), mRNA.	1.06	0.025
2630711	MCM4	Homo sapiens minichromosome maintenance complex component 4 (MCM4), transcript variant 2, mRN	1.06	0.049
5260600	ZNF655	Homo sapiens zinc finger protein 655 (ZNF655), transcript variant 5, mRNA.	1.06	0.048
830397	DKFZP667G2	Homo sapiens hypothetical protein DKFZp667G2110 (DKFZp667G2110), mRNA.	1.06	0.044
3850630	HOMER2	Homo sapiens homer homolog 2 (Drosophila) (HOMER2), transcript variant 1, mRNA.	1.06	0.030
1690719	SYNGR2	Homo sapiens synaptogyrin 2 (SYNGR2), mRNA.	1.06	0.031
3360138	ARL11	Homo sapiens ADP-ribosylation factor-like 11 (ARL11), mRNA.	1.06	0.035
1850674	TRIM31	Homo sapiens tripartite motif-containing 31 (TRIM31), mRNA.	0.90	0.008
4760164	TOR2A	Homo sapiens torsin family 2, member A (TOR2A), mRNA.	0.90	0.007
4610424	RBP4	Homo sapiens retinol binding protein 4, plasma (RBP4), mRNA.	0.90	0.002
830273	FBXL10	Homo sapiens F-box and leucine-rich repeat protein 10 (FBXL10), transcript variant 1, mRNA.	0.90	0.005
7050138	RQCD1	Homo sapiens RCD1 required for cell differentiation1 homolog (S. pombe) (RQCD1), mRNA.	0.89	0.006
130161	LCK	Homo sapiens lymphocyte-specific protein tyrosine kinase (LCK), transcript variant 1, mRNA.	0.89	0.002
4890500	TOR1A	Homo sapiens torsin family 1, member A (torsin A) (TOR1A), mRNA.	0.89	0.007
5560474	RAMP2	Homo sapiens receptor (G protein-coupled) activity modifying protein 2 (RAMP2), mRNA.	0.58	0.007

* Fold changes >0.91 and <1.05 were not considered.
