# Supplementary material for: Short-Term Erythropoietin Treatment Does Not Substantially Modulate Monocyte Transcriptomes of Patients with Combined Heart and Renal Failure
Source: PLoS One. 2012 Sep 5;7(9):e41339. doi: 10.1371/journal.pone.0041339 (PMC3434212; doi:10.1371/journal.pone.0041339)
Supplement: Table S4 — Cardiorenal connector monocyte gene expression panel: changes in CRS patients (n = 12) after 2 weeks of EPO treatment. (PDF) [file pone.0041339.s005.pdf]

Inflammation related genes						Oxidative stress related genes			Renin angiotensin system related genes		
Symbol	Fold change	P-value	Symbol	Fold change	P-value	Symbol	Fold change	P-value	Symbol	Fold change	P-value
IL10	1.06	0.33	CCL26	1.01	0.86	GPX1	0.9410	0.30	ACE	1.0202	0.45
IL10RA	0.96	0.47	CCL27	0.98	0.48	GPX2	1.0121	0.69	AGT	1.0052	0.84
IL10RB	1.02	0.59	CCL3	1.02	0.61	GPX3	0.9932	0.84	AGTR1	1.0586	0.11
IL12RB1	0.95	0.10	CCL3L1	1.05	0.09	GPX4	0.9533	0.38	REN	0.9849	0.61
IL13RA1	0.98	0.73	CCL3L3	1.05	0.32	GPX6	0.9601	0.19	RENBP	1.0178	0.75
IL17C	0.98	0.63	CCL5	1.26	0.04	GPX7	1.0093	0.77			
IL17D	0.96	0.45	CCL8	1.0728	0.07	PRDX1	0.9578	0.40			
IL17E	not expressed	not expressed	CXCL10	1.1044	0.11	PRDX2	1.0135	0.73			
IL17R	1.01	0.89	CXCL12	0.9656	0.33	PRDX3	1.1003	0.11			
IL1F7	0.98	0.54	CXCL14	0.9751	0.38	PRDX4	0.9911	0.84			
IL1R2	0.95	0.37	CXCL16	0.9408	0.19	PRDX5	1.1011	0.16			
IL1RAP	0.99	0.73	CXCL9	not expressed	not expressed	PRDX6	0.9897	0.84			
IL1RL1	0.95	0.13	CXCR4	0.9352	0.15	TXNRD1	0.9384	0.09			
IL1RL1	0.95	0.13	CCR1	0.9736	0.57	TXNRD2	0.9674	0.52			
IL21	0.96	0.11	CCR10	not expressed	not expressed	SELS	1.0724	0.08			
IL21R	0.97	0.30	CCR2	1.0204	0.71	SRXN1	0.9935	0.87			
IL27	0.92	0.10	CCR6	1.0074	0.87	DHRS9	1.0416	0.12			
IL27RA	0.94	0.24	CX3CR1	1.0939	0.17	GSR	1.0154	0.70			
IL2RG	not expressed	not expressed	TLR1	0.9833	0.80	SOD1	0.9307	0.11			
IL4I1	1.03	0.28	TLR10	0.9695	0.27	SOD2	1.0313	0.35			
IL4R	0.98	0.64	TLR2	0.9940	0.88	CCS	1.0252	0.59			
IL5RA	1.00	0.94	TLR4	1.0848	0.20	CYBA	0.9923	0.84			
IL6	not expressed	not expressed	TLR5	1.0369	0.58	DUOX1	-0.0155	0.76			
IL6R	0.97	0.67	TLR6	0.9550	0.15	NCF1	1.0111	0.86			
IL8	0.97	0.78	TLR7	1.0584	0.29	NCF2	0.9849	0.78			
IL8RB	0.98	0.85	TLR8	1.0851	0.29	NOX4	0.9989	0.97			
IFNA10	1.02	0.70	TLR9	1.0288	0.35	NOX5	0.9778	0.41			
IFNA14	1.01	0.67	IRF1	0.9586	0.47	PREX1	0.9242	0.11			
IFNA21	0.98	0.53	IRF3	0.9724	0.58	ALOX15	0.9920	0.78			
IFNA8	0.98	0.68	IRF4	0.9923	0.83	ALOX15B	1.0160	0.66			
IFNAR1	0.95	0.44	IRF5	0.9579	0.41	ALOX5	0.9556	0.45			
IFNAR2	0.95	0.34	IRF7	1.0546	0.32	ALOX5AP	0.9474	0.34			
IFNGR1	1.03	0.61	IRF8	0.9651	0.52	ALOXE3	0.9383	0.09			
IFNGR2	1.04	0.43	CD40	0.9456	0.28	DUSP1	0.9984	0.98			
TNF	0.98	0.71	CD40LG	not expressed	not expressed	MPO	1.0391	0.39			
CCL19	1.01	0.74									
CCL2	1.07	0.13									
CCL21	0.97	0.36									
CCL23	0.96	0.30									
CCL25	0.98	0.43									
